# Thiourea-Based Receptors for Anion Recognition and Signaling

**DOI:** 10.1021/acsomega.3c06861

**Published:** 2024-01-17

**Authors:** Jancarlo Gomez-Vega, Adrian Vasquez-Cornejo, Octavio Juárez-Sánchez, David O. Corona-Martínez, Adrián Ochoa-Terán, Karla A. López-Gastelum, Rogerio R. Sotelo-Mundo, Hisila Santacruz-Ortega, Juan Carlos Gálvez-Ruiz, Refugio Pérez-González, Karen Ochoa Lara

**Affiliations:** † Departamento de Investigación en Polímeros y Materiales, Universidad de Sonora, Rosales y Encinas s/n, Col. Centro CP, 83000 Hermosillo, Sonora, Mexico; ‡ Departamento de Investigación en Física, Universidad de Sonora, Rosales y Encinas s/n, Col. Centro CP, 83000 Hermosillo, Sonora, Mexico; § Departamento de Ciencias Químico Biológicas, Universidad de Sonora, Rosales y Encinas s/n, Col. Centro CP, 83000 Hermosillo, Sonora, Mexico; ∥ Centro de Graduados e Investigación en Química, Instituto Tecnológico de Tijuana, Blvd. Industrial S/N CP, 22510 Tijuana, Baja California, Mexico; ⊥ Laboratorio de Estructura Biomolecular, Centro de Investigación en Alimentación y Desarrollo, A. C., Gustavo Enrique Astiazaran Rosas, No. 46. CP, 83304 Hermosillo, Sonora, Mexico

## Abstract

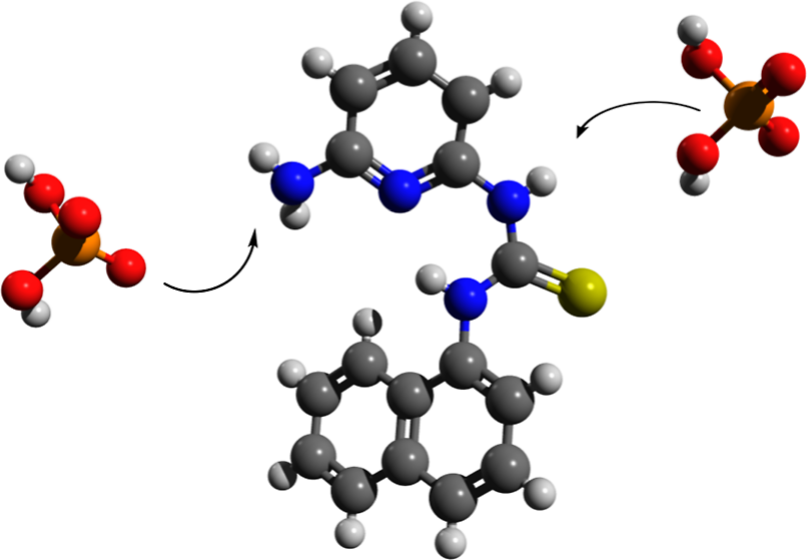

This work reports on two thiourea-based receptors with pyridine and amine units including 1-naphthyl (**MT1N**) and 4-nytrophenyl (**MT4N**) as signaling units. For both compounds, their affinity and signaling ability toward various anions of different geometry and basicity in DMSO were studied using UV–vis, fluorescence, and ^1^H NMR techniques. Anion recognition studies revealed that both **MT1N** and **MT4N** have, in general, high affinities toward basic anions. In this regard, a higher acidity of the **MT4N** receptor was demonstrated. Furthermore, **MT4N** has a higher affinity for fluoride (log *K*_1_ = 5.98) than for the other anions and can effectively detect it through colorimetric changes that can be monitored by the UV–vis technique. The interaction between receptors and anions mainly involves the hydrogens of the amino and thiourea groups of the former. Complementary single-crystal X-ray diffraction studies and molecular modeling at the DFT level were also performed.

## Introduction

During the last decades, anion recognition has been a very important area of supramolecular chemistry that is currently growing.^1,2^ In part, this is because the chemistry involved in this field has diverse applications in different topics such as catalysis,^3,4^ medicine,^5−7^ and materials science, among others.^8−11^ Likewise, anions play a significant role in various environmental processes.^8,12−14^ Therefore, the design of selective and sensitive receptors is a very important issue as well as the implementation of real-time monitoring methods for these species. However, the recognition of anions remains a challenge due to the intrinsic characteristics of these species, such as geometry, small charge density, high solvation energies, and possible pH dependence, among others.^15,16^ In this context, in recent decades various approaches to anion recognition and signaling have been addressed, the most common involving hydrogen bond donor groups as receptor units,^17^ such as amides,^18,19^ ureas,^20,21^ thioureas,^20,22^ squaramides,^23−25^ and some combinations of the previous ones,^26,27^ among others. This approach is based on taking advantage of the directionality and additivity that hydrogen bonding (HB) can confer. Furthermore, these units can be modulated to increase their acidity simply by attaching them to electron-withdrawing groups, which also enhances their HB-donor character. However, it has been shown that this strategy does not always imply an increase in the affinity between the receptor and the guest, so the incorporation of this type of groups should be carefully considered.^2,28^ It is also important to note that by increasing the acidity of the receptor units, the presence of sufficiently basic anions such as fluoride or acetate can turn the recognition process into a typical acid–base reaction.^29^ On the other hand, to signal the process of molecular recognition, groups of chromophores and fluorophores are frequently incorporated into the receptor, which serve for the transduction of the recognition process, or where applicable, the acid–base process triggered by the presence of the anion.^30^ It should be mentioned that this general strategy, especially for thiourea-type receptors, is still valid as shown in the recent review by Al-Saidi and Khan;^22^ this is because it offers the possibility of obtaining colorimetric and fluorescent chemosensors for anions of diverse nature, even in highly competitive environments. In addition, it is worth mentioning that the works of Fabbrizzi and Amendola,^21,23,29,31^ Gunnlaugsson^28,32^ and Yatsimirsky^33^ have established a good starting point for understanding receptor–guest systems that involve acid–base equilibria.

**Figure 1:**
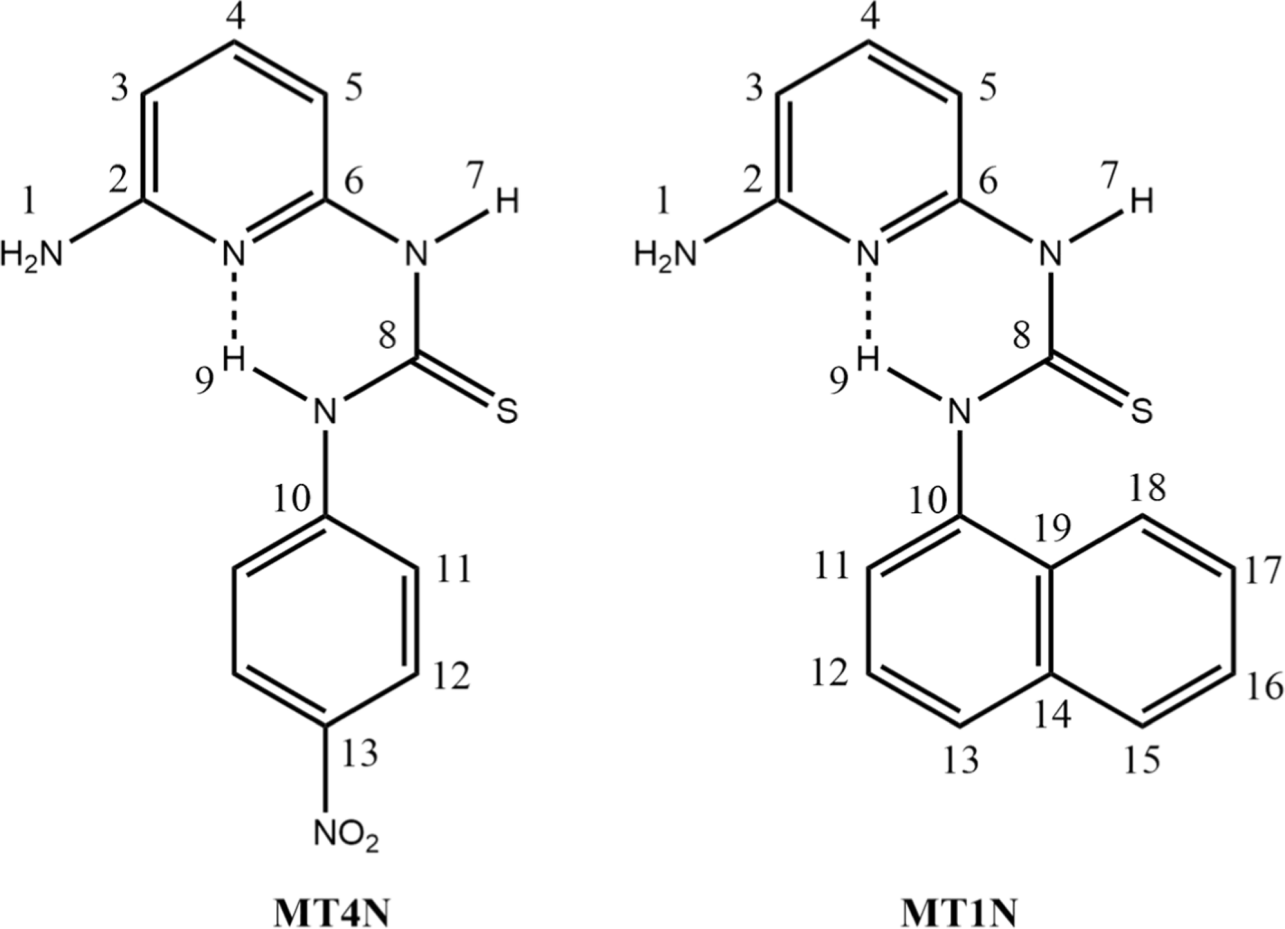
Chemical structures of the **MT4N** and **MT1N** receptors studied in this work.

Considering all the above, this work aims to contribute to the state of the art by evaluating two thiourea-type receptors in the recognition and signaling of various anions. The structure of the receptors consists of a thiourea group covalently attached to a fluorophore (**MT1N**) or a chromophore (**MT4N**), with an aminopyridine group attached to the thiourea group on the other side (see Figure 1). This design is based on the idea that the aromatic substituents enhance the bond with the anions;^28^ in addition, they can delocalize the charge generated in case of deprotonation, thus producing signaling in UV–vis^32^ or fluorescence.^31^ It is important to mention that the **MT1N** receptor represents a new compound in the literature, while the **MT4N** receptor has been previously reported.^34,35^ However, anion recognition studies with the latter have not been reported.

## Experimental Section

### General Procedures and Materials

All reagents and solvents employed for the synthesis and molecular recognition studies were purchased from commercial suppliers and used without further purification.

### Instruments

^1^H NMR studies were carried out on a Bruker AVANCE III 400 spectrometer, and standard references were used: tetramethylsilane (TMS) (δ ^1^H (400 MHz) = 0 and δ ^13^C (100 MHz) = 0). IR spectra were recorded on a PerkinElmer FT-IR/FIR Spectrometer model FRONTIER instrument using attenuated total reflection (ATR). Mass spectrometry was conducted on an Agilent 6100 LC/MS using the ESI^+^ mode. The titrations by UV–vis were performed on an Agilent 8435 ultraviolet–visible spectrophotometer (Agilent Technologies) equipped with a deuterium and halogen lamp, using a spectral range of 187 to 1100 nm and quartz cells with a 1 cm optical path length. Fluorescence studies were carried out on a Lambda LB-50 with a xenon lamp, with quantifications consisting of 1–1000 light pulses per measurement, depending on the required reading quality and speed, and with a glass cell with a 1 cm light path. Melting points were recorded on a Büchi melting point B-545 apparatus.

### Solution Studies

As a part of the preliminary investigations, the electronic absorption and emission spectra were measured within a range of 10^–6^ to 10^–5^ M in DMSO. From these data, spectral characteristics of the compounds such as maximum wavelengths, molar extinction coefficients (ε), and excitation and emission maximum wavelengths were obtained. On the other hand, the molecular recognition studies of the receptors were performed by UV–vis and ^1^H NMR with the anions F^–^, Cl^–^, C_6_H_5_CO_2_^–^, CH_3_CO_2_^–^, NO_3_^–^, HSO_4_^–^, and H_2_PO_4_^–^ in their tetrabutylammonium salt forms. The titrations were carried out following the typical procedure where the receptor concentration is kept constant (3 × 10^–5^ and 3.6 × 10^–5^ M for **MT1N** and **MT4N**, respectively, when using UV–vis and 3 × 10^–3^ M for both receptors when NMR was used). Each titration was performed at least in triplicate at 298 K. Only for the case of **MT1N**, calibration curves were performed by the batch method in DMSO, at least in triplicate, using the fluorescence technique maintaining a constant receptor concentration of 1 × 10^–5^ M.

### Data Analysis

The fitting of the data obtained by UV–vis was carried out starting from the matrix form of Lambert–Beer’s law (eq 1) and the least-squares method.
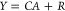
1

where *Y* corresponds to the matrix of measured absorbances, *C* is the matrix of concentrations of the absorbing species, *A* is the matrix of molar absorptivities, and R is the matrix of residuals or intrinsic errors in the analytical measurements. To minimize the residuals of eq 1, the steps described by Maeder and Zuberbuehler in 1990^36^ have been followed. For this, first, the linear parameters (*A*) were eliminated from the equation and the data from the *Y* matrix were reduced using singular value decomposition (SVD), thus obtaining the *Y*′ matrix, which is a projection of the original *Y* matrix. In this way, the objective function to minimize is the one described in eq 2.
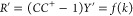
2

where *R*′ corresponds to the projection of the residuals matrix, C^+^ is the Moore–Penrose pseudoinverse of the concentrations matrix, *Y*′ is the projection of the absorbance matrix, and *f*(*k*) indicates that this function is a function that only depends on the nonlinear parameters *k* (association constants) since the concentration matrix *C* only depends on the association constants. It is important to note that the *C* matrix was obtained using equilibrium models based on the law of mass action through the Newton–Raphson or Levenberg–Marquardt algorithms previously reported by Maeder.^37^ Finally, for the implementation of the calculations and the minimization of eq 2, Python 3.11.1 and the minimization tools available in the Scipy 1.10.1 module were used.^38^

The fitting of the ^1^H NMR data was performed using the same computational tools mentioned above and the same methodology for obtaining the concentration matrix *C*. The essential difference in this case is that the observed property is dependent on the molar fraction and not directly on the concentration as shown by eq 3.
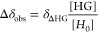
3

where Δδ_obs_ are the observed chemical shifts in ppm, δ_ΔHG_ is the complexation-induced chemical shift, [HG] is the concentration of the receptor (H)–guest (G) complex, [H_0_] is the initial concentration of the receptor. Therefore, using the linear relationship described in eq 3 and redefining the molar fraction ([HG]/[H_0_]) for each species as the matrix *X* and Δδ_obs_ as a matrix of observed chemical shifts, then the function to minimize is the sum of squares (ssq) as shown in eq 5.
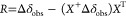
4
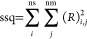
5

where ns is the number of samples, nm is the number of measurements, and *X*^+^ and *X*^T^ refer to the pseudoinverse and the transposition of the molar fraction matrix of the species in solution, respectively.

The methods described above for UV–vis and ^1^H NMR were used to adjust conventional 1:1 and 1:2 receptor–guest interactions and the following chemical equations
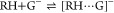
6
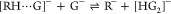
7
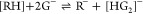
8

where RH schematizes the protonated receptors and G^–^ are the anions.

For the weighted linear regression analysis by fluorescence, the methodology described by Miller and Miller in 2010 was used.^39^

### Preparative Part

#### Synthesis of Receptor **MT4N**

Receptor **MT4N** was prepared according to the following procedure: 0.424 g (2.35 mmol) of 4-nitrophenyl isothiocyanate dissolved in 10 mL of anhydrous acetonitrile were added to a solution of 0.205 g (1.88 mmol) of 2,6-diaminopyridine in the same solvent (15 mL), maintaining an inert atmosphere of N_2_. A yellow precipitate was observed after the mixture was stirred over 24 h at room temperature. The solid was filtered and washed with acetonitrile. This product was then recrystallized from acetone and dried in vacuo. Yield: 0.483 g (89%). mp 197.9–198.7 °C. UV absorption: λ_max_ (DMSO)/355 nm (ε/dm^3^ mol^–1^ cm^–1^ = 14,624 ± 121). FT-IR (ATR): 3480, 3356, 1568, 1538, 1505, 1450, 1340, 1320, 1296, 1266 cm^–1^. ^1^H NMR (400 MHz, DMSO-*d*_6,_ δ, ppm): δ 14.42 (s, 1H, H-9), 10.78 (d, *J* = 5.5 Hz, 1H, H-7), 8.23 (d, *J* = 5.3 Hz, 4H, H-11 & H-12), 7.42 (t, *J* = 8.0 Hz, 1H, H-4), 6.50 (s, 2H, H-1), 6.36 (d, *J* = 7.7 Hz, 1H, H-5), 6.18 (d, *J* = 8.1 Hz, 1H, H-3). ^13^C NMR (101 MHz, DMSO, ppm): 178.2 (C-8), 157.6 (C-2), 152.3 (C-6), 145.8 (C-13), 143.8 (C-10), 140.6 (C-4), 124.4 (C-12), 123.8 (C-11), 102.8 (C-5), 99.6 (C-3). MS-ESI (+) *m*/*z*: 291.68 [M + H]^+^ (100%). Elem. Anal. Calcd for C_12_H_11_N_5_O_2_S: C, 49.82; H, 3.83; N, 24.21; O, 11.06; S, 11.08. Found: C, 49.56; H, 3.61; N, 23.86; S, 10.69.

#### Synthesis of Receptor **MT1N**

Receptor **MT1N** was prepared according to the following procedure: 0.438 g (2.365 mmol) of 1-naphthyl isothiocyanate dissolved in 10 mL of anhydrous acetonitrile was added to a solution of 0.205 g (1.880 mmol) of 2,6-diaminopyridine in the same solvent (17 mL), maintaining an inert atmosphere of N_2_. A gray-brownish precipitate was observed after the mixture was stirred over 48 h at room temperature. The solid was filtered and washed with acetonitrile. This product was then recrystallized from acetone and dried in vacuo. Yield: 0.48 g (87%). mp 213.2–214.6 °C. UV absorption: λ_max_ (DMSO)/331 nm (ε/dm^3^ mol^–1^ cm^–1^ = 15,578 ± 110). FT-IR (ATR) cm^–1^: 3453, 3346, 1449, 1530, 1505, 1253, 1234, 1197, 772, 724. ^1^H NMR (400 MHz, DMSO-*d*_6,_ δ, ppm): δ 13.62 (s, 1H, H-9), 10.59 (s, 1H, H-7), hydrogen atoms of the naphthalene unit gave a series of signals in the range from 7.5 to 8.03 ppm that integrate for 7 hydrogen atoms. 7.42 (t, *J* = 8.0 Hz, 1H, H-4), 6.38 (dd, *J* = 7.8, 0.7 Hz, 1H, H-5), 6.30 (s, 1H, H-1), 6.13 (dd, *J* = 8.1, 0.7 Hz, 1H, H-3). ^13^C NMR (101 MHz, DMSO, ppm): δ 180.9 (C-8), 157.9 (C-2), 153.0 (C-6), 140.3 (C-4), 136.5 (C-10), 134.3 (C-14), 130.5 (C-19), 128.5 (C-15), 127.1 (C-16), 126.7 (C-12), 126.5 (C-17), 126.1 (C-13), 125.9 (C-18) 123.7 (C-11), 101.8 (C-5), 99.1 (C-3). MS-ESI (+) *m*/*z*: 296.79 [M + H]^+^ (100%).

### X-ray Crystallography

Single crystals of **MT1N** and **MT4N** were both obtained by slow evaporation of the solvent at room temperature from supersaturated solutions in acetone. The single crystals were mounted on glass fiber and studied with a Bruker D8 QUEST diffractometer system equipped with a Multilayer mirror monochromator and a Microfocus sealed tube (Cu Kα). The crystals were kept at 273(2) K during data collection. Structure solution and refinement were carried out with the Olex2,^40^ SHELXT,^41^ and SHELXL^42^ software used to prepare material for publication. Figures have been created with Mercury.^43^ Selected crystallographic data for **MT1N** and **MT4N** are shown in Table 1. Crystallographic data for the structure reported in this article (**MT1N**) have been deposited at the Cambridge Crystallographic Data Centre as supplementary publication no. 2291426. Copies of the data can be obtained free of charge by application to CCDC, 12 Union Road, Cambridge, CB2 1EZ, UK (fax: (+44) 1223-336-033, e-mail: deposit@ccdc.cam.ac.uk).

**Table 1:** Crystal Data and Structure Refinement

identification code	**MT1N**	**MT4N**
empirical formula	C_16_H_14_N_4_S	C_12_H_11_N_5_O_2_S
formula weight	312.1	289.32
temperature/K	273.15	273.15
crystal system	triclinic	monoclinic
space group	*P*1̅	*P*2_1_/*n*
*a*/Å	8.0722(2)	10.8420(4)
*b*/Å	9.2290(3)	10.4179(3)
*c*/Å	10.7553(3)	12.3012(4)
α/deg	68.902(2)	90
β/deg	81.656(2)	110.4780(10)
γ/deg	72.161(2)	90
volume/Å^3^	711.05(4)	1301.63(7)
*Z*	2	4
ρ_calc_g/cm^3^	1.458	1.476
μ/mm–^1^	2.877	2.314
*F*(000)	325	600
crystal size/mm^3^	0.51 × 0.214 × 0.064	0.347 × 0.183 × 0.042
radiation	Cu Kα (λ = 1.54178)	Cu Kα (λ = 1.54178)
2Θ range for data collection/deg	8.82 to 129.726	9.378 to 124.04
index ranges	–9 ≤ *h* ≤ 9, –10 ≤ *k* ≤ 10, –12 ≤ l ≤ 12	–12 ≤ *h* ≤ 12, –11 ≤ *k* ≤ 11, –14 ≤ *l* ≤ 13
reflections collected	18,579	18,636
independent reflections	2252 [*R*_int_ = 0.0649, *R*_sigma_ = 0.0357]	2037 [*R*_int_ = 0.0495, *R*_sigma_ = 0.0273]
data/restraints/parameters	2252/0/192	2037/0/183
goodness-of-fit on *F*^2^	1.032	1.17
final *R* indexes [*I* ≥ 2σ(*I*)]	*R*_1_ = 0.0368, w*R*_2_ = 0.0888	*R*1 = 0.0666, wR2 = 0.1467
final *R* indexes [all data]	*R*_1_ = 0.0564, w*R*_2_ = 0.0983	*R*_1_ = 0.0749, w*R*_2_ = 0.1565
largest diff. peak/hole/e Å–^3^	0.17/–0.18	0.63/–0.70

### Molecular Modeling

For molecular modeling studies, the initial molecular structures of the **MT4N** and **MT1N** receptors were constructed using their X-ray diffraction (XRD) coordinates and then geometrically optimized using density functional theory implemented in the Gaussian 09 program package.^44^ The chemical model used was B3LYP/6-31G* using DMSO as solvent. Once optimized, they were used to form their respective chloride and acetate complexes. Their frequencies were calculated to define if the resulting structures were a local minimum or a transition state. All these calculations were performed with the Gaussian 09 (version B.01) software package^44^ using the ACARUS (High-Performance Computing Area of the University of Sonora) high-performance cluster and the molecular structures were visualized with the Avogadro program.^45^

## Results and Discussion

### Synthesis

**Scheme 1:**
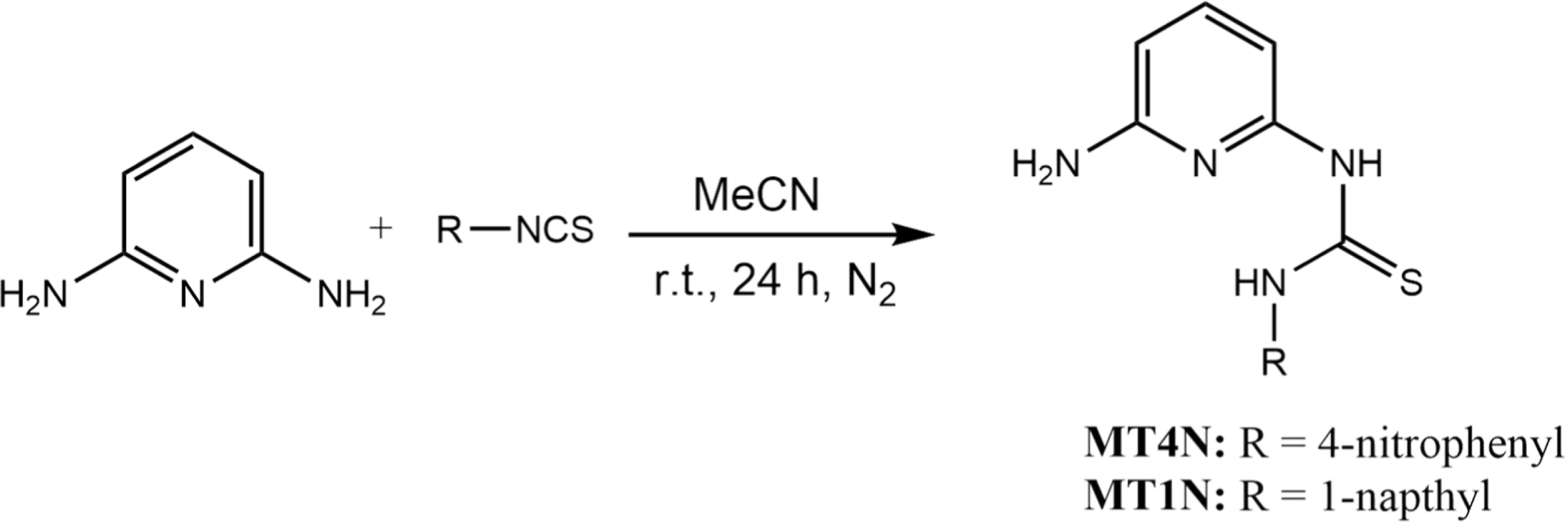
Synthesis of the **MT4N** and **MT1N** Receptors

The two bis-thiourea type receptors were synthesized as described in Scheme 1.

Both receptors were obtained with good yields, approximately 90%. In this context, it is important to highlight that the previously described synthetic methodology for obtaining **MT4N** differs from the one reported here and with a lower yield.^34^ The receptors were characterized by FT-IR and ^1^H and ^13^C NMR (Figures S1–S6), elemental analysis, and single-crystal XRD. In addition, they were characterized in solution by UV–vis and fluorescence. In all cases, the observed spectroscopic data are consistent with the proposed structures.

First, the formation of the thiourea receptors was confirmed by ^1^H NMR spectra, which shows the signals of the thioureic hydrogens –NH (H-9 and H-7) as singlets at δ = 14.44 ppm (H-9) and 10.81 ppm (H-7) in the case of **MT4N**, and at δ = 13.62 ppm (H-9) and 10.59 ppm (H-7) for **MT1N**. These data demonstrate that the hydrogens H-9 and H-7 of the thiourea unit of **MT4N** are downfield than those of **MT1N**, which can be attributed to the presence of the electron-withdrawing –NO_2_ group; consequently, they are more acidic in **MT4N**. Moreover, in both receptors, it was observed that H-9 is downfield compared to H-7. This can be explained by the proximity of H-9 to the chromophore groups, whose ability to delocalize the electron density induces a deprotection effect on this proton. Likewise, the signal of the amino group –NH_2_ (H-1) appears as a broad singlet at δ = 6.52 and 6.30 ppm, respectively, for **MT4N** and **MT1N**. This slight difference in the chemical shift of H-1 of **MT4N** compared to **MT1N** is possibly due to the influence of an intramolecular hydrogen bond between H-9 and the nitrogen of the pyridine ring. Such interaction has been previously reported in the solid state for **MT4N** and similar systems (and described in the single-crystal XRD results).^34^ On the other hand, in the ^13^C NMR spectra, the signals of the carbons of the thiocarbonyl group are at δ = 178.18 and 180.88 ppm for **MT4N** and **MT1N**, respectively.

Regarding the FT-IR spectra, the stretching of the N_2_C=S bonds is observed at 1253, 1234, and 1197 cm^–1^ for **MT1N**, while the corresponding one for **MT4N** is observed at 1320, 1296, and 1266 cm^–1^. Characteristic bands of the monosubstituted naphthyl group belonging to the CH bending of the aromatic rings are observed at 772 and 724 cm^–1^ for **MT1N**. In contrast, **MT4N** shows bands at 1568 and 1340 cm^–1^, characteristic of the in-phase and out-of-phase stretching of the bonds of the NO_2_ group.

**Figure 2:**
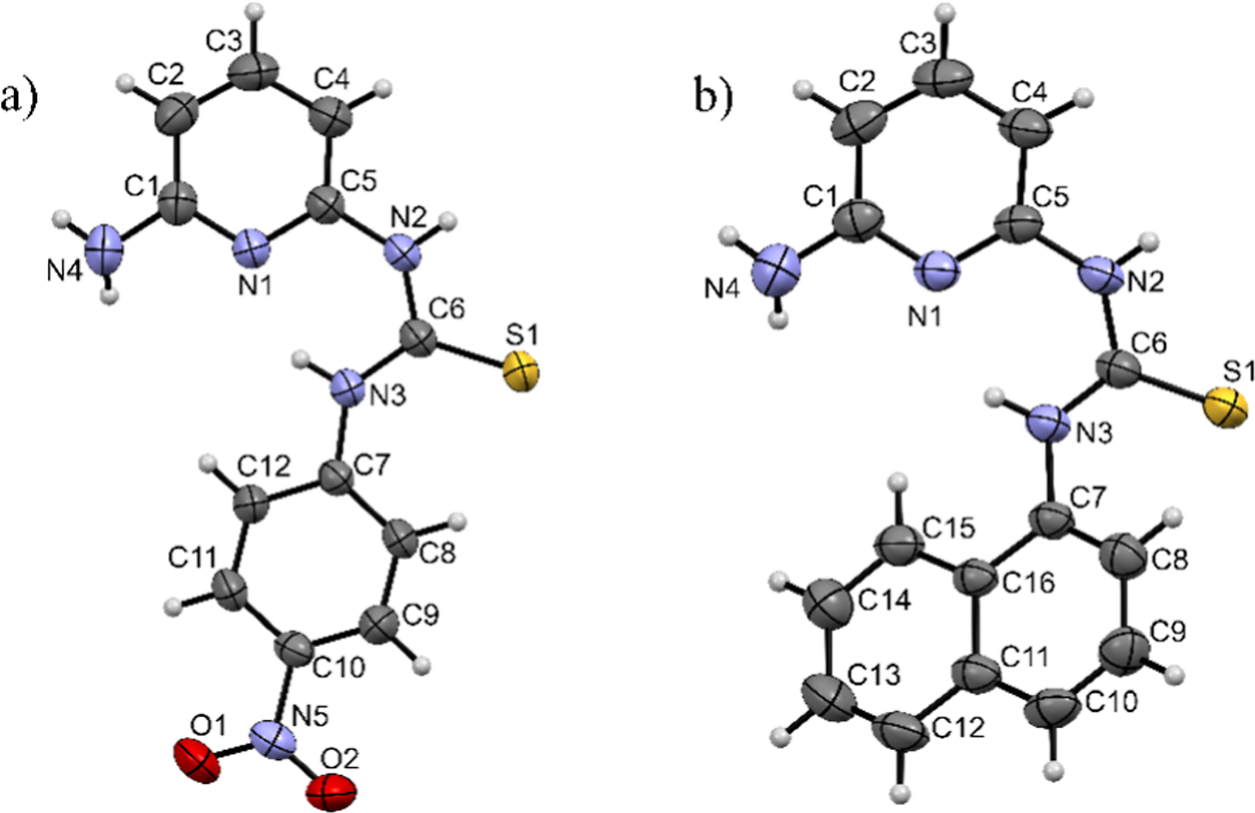
ORTEP drawings with atom numbering scheme and displacement ellipsoids at 50% probability for (a) **MT4N** and (b) **MT1N**.

Additionally, the structures of **MT4N** and **MT1N** were confirmed by single-crystal XRD, as shown in Figure 2. The structural features elucidated by XRD for **MT4N** reveal crystallographic parameters highly similar to those previously reported for this compound by the group of Kelman et al. 2003.^34^ Among these features, three are primarily noteworthy. The first is the planarity of the molecule, which was measured by calculating the angle between the mean planes of the two aromatic rings. For **MT4N**, an angle of 9.37° was obtained, whereas the previously reported structure for this molecule exhibits a slightly flatter conformation, with an angle of 9.06°.^34^ In contrast, the angle of the **MT1N** structure is 38.54°, indicating that the conformation of this molecule is not planar. The second significant structural aspect is the observation of the presence of an intramolecular hydrogen bond between the atom of thiourea group N3 and nitrogen N1 of the pyridine ring, observed in both receptors. The distances for this hydrogen bond (N1···H–N3) are as follows: in the case of **MT4N**, the N1···H interaction has a distance of 1.894 (2) Å and the H–N bond a distance of 0.860 (2) Å, with an angle N1···H–N3 of 144.39° (15). For the **MT1N** receptor, a distance N1···H of 2.005 (17) Å and a distance H–N = 0.860 (2) Å were observed, with an angle of 138.03° (10). Considering the angle and the observed distances, we can infer that the interaction of the intramolecular hydrogen bond is stronger in **MT4N** compared to **MT1N**. This information also supports the previously mentioned hypothesis for the ^1^H NMR data regarding the influence of the intramolecular hydrogen bond on the observed chemical shift in the protons of the amino group. Finally, the third relevant feature of the crystal structures of the receptors is the existence of two available sites for donating hydrogen bonds: the amino group of the pyridine ring and the thiourea hydrogen N2–H. This latter observation also applies in solution, as demonstrated by NMR on the NOESY spectra shown in Figures S7 and S8.

### Solution Studies

#### UV–vis

Molecular recognition studies were performed using the UV–vis electronic absorption technique at 25 °C and DMSO as solvent. Under these conditions, the spectrum of **MT4N** displays two absorption bands with maxima at 277 and 355 nm (Figure S9). The spectrum of **MT1N** exhibits two absorption bands of similar intensity with maxima at 272 and 331 nm (Figure S10). These absorption bands for both receptors correspond to transitions of the π → π* type.

**Figure 3:**
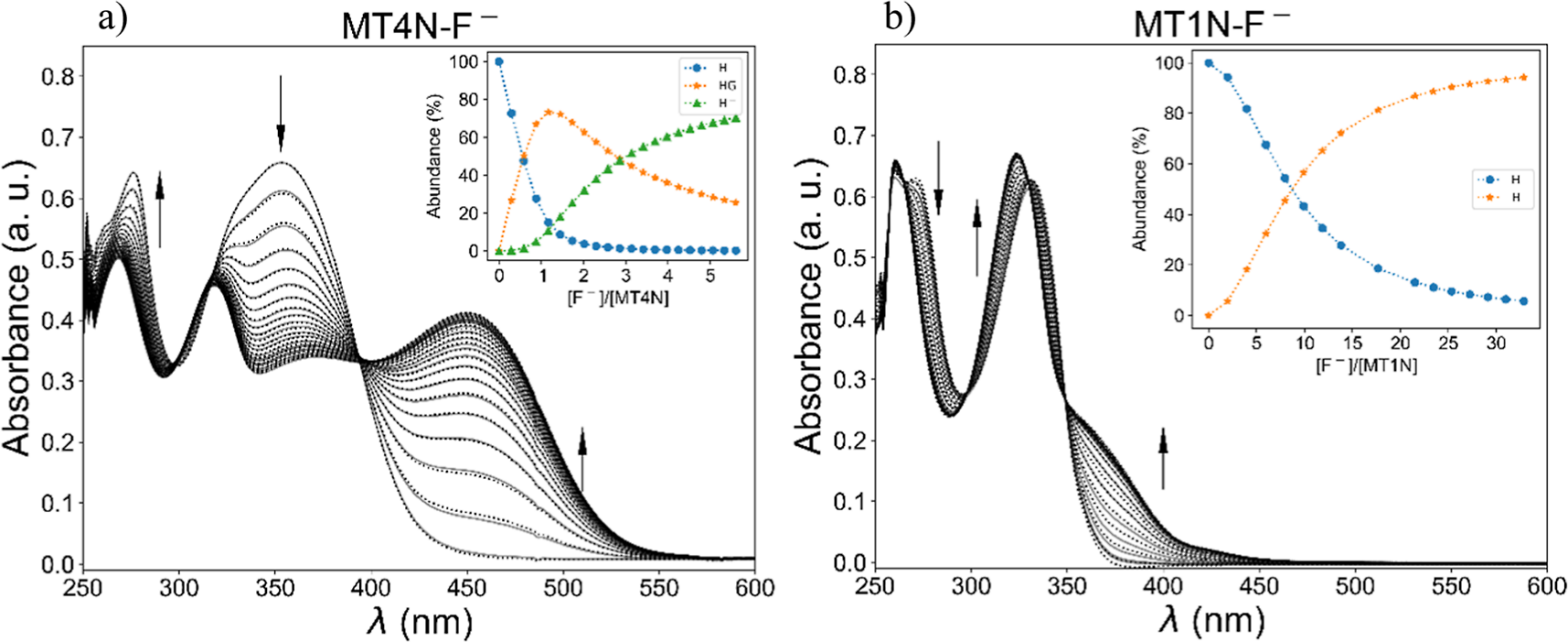
Absorption spectra of receptors with increasing concentration of F^–^ in DMSO at 298 K. The top right inset shows the relative abundance of the different species during the titration, where H = receptor and G = anion guest. The dashed lines represent the theoretical profiles obtained from data fitting. (a) [**MT4N**] = 3.6 × 10^–5^ M with F^–^ (0–1.45 × 10^–4^ M); (b) [**MT1N**] = 3 × 10^–5^ M with F^–^ (0–9.87 × 10^–4^ M).

On the other hand, titrations were performed with the receptors and the anions F^–^, Cl^–^, CH_3_CO_2_^–^, H_2_PO_4_^–^, HSO_4_^–^, and NO_3_^–^. In the case of the **MT4N**–HSO_4_^–^ and **MT1N**–H_2_PO_4_^–^ systems, the observed changes in the absorption spectra of the receptors, induced by complexation, were very small and, therefore, their experimental reproduction was not feasible. Regarding the rest of the complexes, Figure 3 presents, as a representative example, the absorption spectra obtained in the titrations of **MT4N** and **MT1N** with the fluoride anion. Several isosbestic points are observed in these spectra as well as the appearance of a charge-transfer band at 455 nm for **MT4N** and at 380 nm for **MT1N**.

Due to the structural design of both receptors, the observation of the charge-transfer band in the presence of fluoride is an expected phenomenon, which can be attributed to the deprotonation of the receptors by the basic anion, which causes a noticeable color change in the solution, going from a colorless solution to a yellow solution (Figure S11). In the case of **MT1N**, this color change is less evident (Figure S11b), compared to the corresponding change observed for **MT4N** (Figure S11a). However, under ultraviolet light (λ_ex_ = 365 nm), the response of **MT1N** in the presence of F^–^ and other basic anions is quite evident (Figure S11c). This colorimetric response, like the one described above, has been widely reported in the literature for receptors analogous to those reported in this study and is, therefore, a common strategy for fluoride signaling.^22,44^

It is important to note that the UV–vis response of **MT1N** in the presence of the other anions was considerably different from that observed with fluoride (see Figures S12–S16), as the charge-transfer band related to deprotonation was not observed in these systems. In the case of titrations involving the **MT4N** receptor, its spectral behavior in the presence of the anions Cl^–^, NO_3_^–^, and HSO_4_^–^ was also markedly different from that observed with fluoride (see Figures 3 and S17–S19). Meanwhile, with the anions H_2_PO_4_^–^ and CH_3_CO_2_^–^, the results indicate that these anions can deprotonate the receptor, as evidenced by the appearance of a charge-transfer band of lower intensity (see Figures S20 and S21). From the data obtained in the titrations, it was possible to calculate the association constants for the receptor–anion systems, which are summarized in Table 2.

**Table 2:** Logarithmic Binding Constants (log_10_(*K*)) for Complexes between Receptors and Diverse Anions Obtained by UV–vis in DMSO at 298 K

system	log_10_(***K***)	F^–^	CH_3_CO_2_^–^	H_2_PO_4_^–^	Cl^–^	NO_3_^–^	HSO_4_^–^
**MT1N**	log_10_(*K*_1_)		3.13 (0.20)	d	1.80 (0.12)	1.91 (0.01)	1.94 (0.26)
	log_10_(*K*_2_)		2.53 (0.20)				
	log_10_(β)	7.28 (0.06)^a^	5.66^c^				
**MT4N**	log_10_(*K*_1_)	5.98 (0.35)	4.07 (0.27)	1.60 (0.02)	1.92 (0.03)	1.72 (0.01)	d
	log_10_(*K*_2_)	4.35 (0.11)	4.23 (0.21)	5.07 (0.31)			
	log_10_(β)	9.84^b^	8.30	6.47			

a Constant obtained from the global reaction described by eq 8. Therefore, it is referred to as the global constant (β).

b Eqs 6 and 7 fitted by the stepwise binding model.

c Noncooperative 1:2 host–guest model.^45^

d Not determined due to poor observed changes.

From the data in Table 2, it can be seen that most of the systems involve two equilibria. The least-squares fitting of the data for the **MT4N** systems with F^–^, CH_3_CO_2_^–^ and H_2_PO_4_^–^ was performed considering the successive equilibria described in eqs 5 and 6. On the other hand, the **MT1N**–F^–^ system is best described by the fitting of the global eq 7. In this regard, it was observed that the main difference between these two types of fitting is that the successive fitting with eqs 5 and 6 works when log_10_(*K*_1_) has a magnitude comparable to that of log_10_(*K*_2_); otherwise, the interaction between the receptor and the guest is governed by the equilibrium of eq 7, that is, the deprotonation of the receptor.

Regarding the log_10_(β) values observed for the aforementioned systems, **MT4N** generally exhibits higher magnitudes for the constants compared to those corresponding to **MT1N**. A trend is also observed for both receptors in the log_10_(β) values with respect to the anions in the order F^–^ > CH_3_CO_2_^–^ > H_2_PO_4_^–^. Moreover, the systems between the **MT4N** receptor and the anions F^–^, CH_3_CO_2_^–^, and H_2_PO_4_^–^ exhibit log_10_(*K*_1_) values in the same decreasing order mentioned, thereby indicating that this receptor forms hydrogen bonds with the anions prior to undergoing deprotonation. In this context, when log_10_(*K*_1_) decreases log_10_(*K*_2_) increases, meaning that the stronger the hydrogen bond, the less favored the deprotonation. This statement is consistent with what was previously described by Pérez-Casas and Yatsimirsky in 2007.^33^ Likewise, it can be inferred that the geometry and basicity of the studied anions are important factors in the affinity shown by these receptors.

On the other hand, the data from titration of **MT1N**–CH_3_CO_2_^–^ system were fitted to a 1:2 noncooperative host–guest model, as previously described by the Thordarson group.^45−47^ In particular, the absence of the charge-transfer band in the **MT1N**–CH_3_CO_2_^–^ complex can be considered evidence of the lower acidity of the **MT1N** receptor compared to its analog **MT4N**. Finally, the **MT4N**–NO_3_^–^ and **MT4N**–Cl^–^ system as well the **MT1N** complexes with Cl^–^, NO_3_^–^, and HSO_4_^–^ were fitted considering a 1:1 receptor–guest equilibrium. However, the changes observed in these experiments were small and very close to the lower limit of quantification (LOQ) by this technique. Nevertheless, as these are reproducible experiments, they can be taken as evidence that the interaction between the receptors and these anions does not induce sufficient electronic changes in the chromophores; therefore, signaling the complexation phenomenon using this technique is not most appropriate.

#### ^1^H NMR

**Figure 4:**
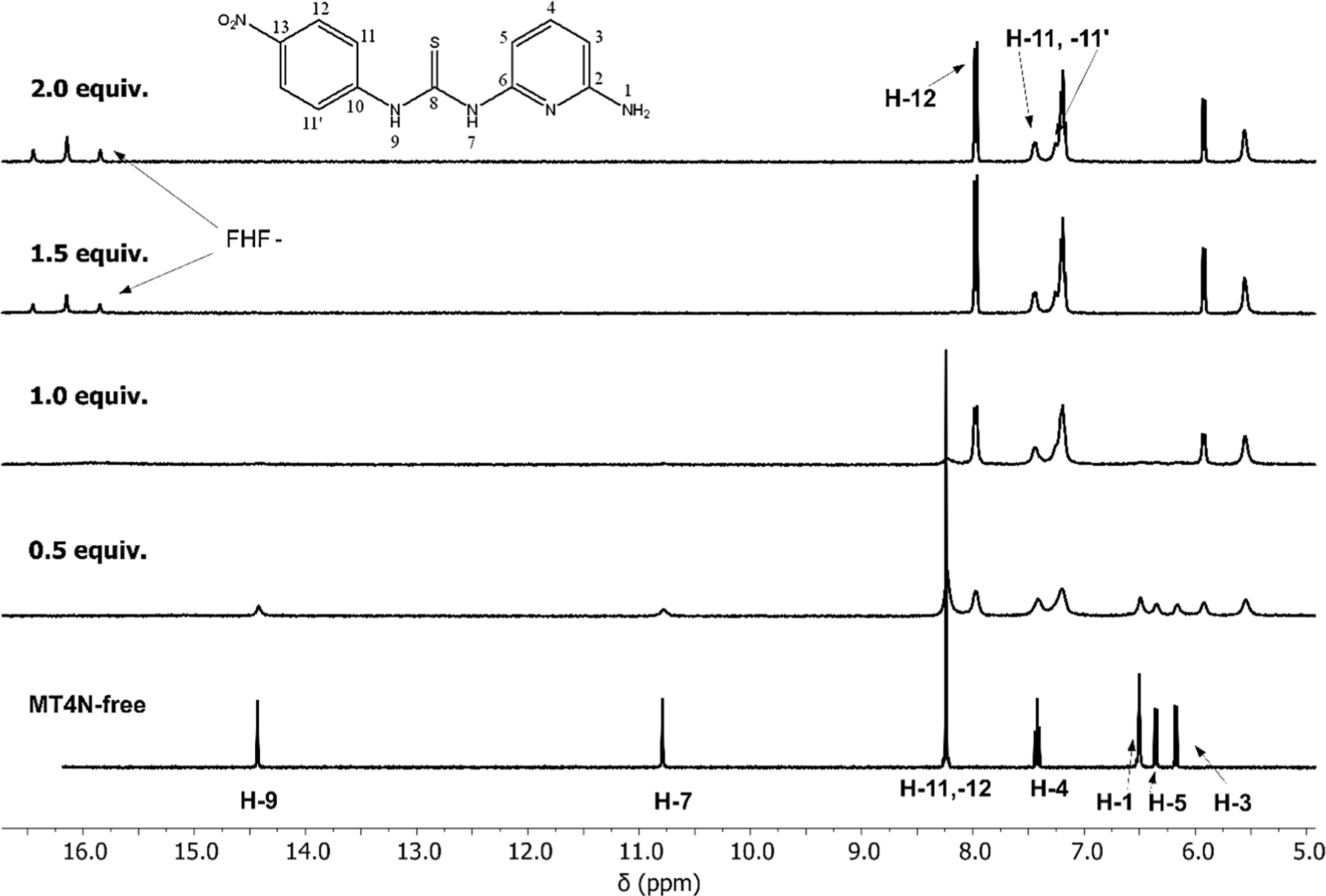
^1^H NMR spectra of **MT4N** (3 mM) with increasing concentration of F^–^ (0–6 × 10^–3^ M) in DMSO-*d*_6_ at 298 K.

To deepen the understanding of the various receptor–anion systems, complexation studies were performed using the ^1^H NMR technique. In this regard, the ^1^H NMR spectra of receptor **MT4N** in the presence of the anions F^–^, CH_3_CO_2_^–^, and H_2_PO_4_^–^, evidenced the deprotonation process, which agrees with the charge-transfer band observed for the receptor by UV–vis in these systems (Figures 4, S22, and S23). Specifically, the clearest evidence was observed with the F- anion, since during the titration a triplet centered at 16.14 ppm appeared, corresponding to the FHF^–^ species, see Figure 4. In this case, an upfield shift of the H-1 signal (amino group) after deprotonation, from 6.50 to 5.56 ppm, is also noteworthy. This phenomenon can be attributed to the breaking of the intramolecular hydrogen bond (see Figure 2), as the chemical shift of H-1 is influenced by this interaction.

On the other hand, the signals of the protons H-11 and H-12 appear as a singlet of high intensity at 8.24 ppm, but upon the addition of two equiv of F-, they are shifted upfield and split due to electronic rearrangement following deprotonation. This behavior is due to the loss of symmetry and chemical equivalence between these protons, causing the new signals to appear as a doublet for H-12, centered at 7.97 ppm, while H-11 splits into a double doublet, with a signal centered at 7.44 ppm (H-11) and H-11′ at 7.25 ppm. The protons H-3 and H-4 undergo an upfield shift following the addition of F-, while the signal for H-5 shifts downfield, falling within the set of signals for the protons H-4 and H-11′ at approximately 7.20 ppm.

The pattern of signals previously described for the **MT4N**–F^–^ system is similar to those observed for the systems of this receptor and the anions CH_3_CO_2_^–^ and H_2_PO_4_^–^, although to a lesser extent. Additionally, for these systems, a significant broadening of the signals belonging to the HB-donating protons (H-9, H-7, and H-1) was observed, which prevented reliable monitoring of their chemical shifts (Figures S22 and S23). With respect to the spectra of **MT1N** in the presence of F^–^, it was observed that the protons H-1-H-5, H-7, and H-9 of the receptor showed very similar behavior to that described for the **MT4N**–F^–^ system (Figure S27); although in this case, the appearance of the FHF^–^ dimer was evidenced with twice the equivalents of F^–^. On the other hand, it is important to highlight that in the experiments of **MT1N** with CH_3_CO_2_^–^ and H_2_PO_4_^–^, no evidence of receptor deprotonation was observed (Figures S29 and S30), indicating a lower acidity of **MT1N** compared to **MT4N**.

Furthermore, from the data from NMR titrations, it was possible to study the complexation process of the systems formed between **MT1N** and the anions CH_3_CO_2_^–^, H_2_PO_4_^–^, C_6_H_5_CO_2_^–^, and Cl^–^ (Figures S28–S30 and S32), while with **MT4N** this was only possible with C_6_H_5_CO_2_^–^ and Cl^–^ (Figures S24 and S25). From all these experiments, it can be demonstrated that the protons primarily involved in the complexation process are H-7, H-1, and to a lesser extent, H-9. This suggests that the participation of the thiourea protons in the formation of a hydrogen bond with the anion is not simultaneous, probably due to the intramolecular hydrogen bond of H-9 with the nitrogen of the pyridine ring. Moreover, given the conformation suggested by the crystal structure, as well as computational models, it is evident that the formation of the complexes is consistent with a 1:2 receptor–host model. Considering the above, least-squares fitting was made to the data obtained from the titrations to determine the association constants; these values are summarized in Table 3.

**Table 3:** Logarithmic Binding Constants (log_10_(*K*)) for Complexes between Receptors and Diverse Anions Obtained by ^1^H NMR in DMSO-*d*_6_ at 298 K

system	log_10_(***K***)	F^–^	CH_3_CO_2_^–^	H_2_PO_4_^–^	C_6_H_5_CO_2_^–^	HSO_4_^–^	Cl^–^
**MT1N**	log_10_(*K*_1_)	b	2.79 (0.07)	4.56 (0.54)	4.06 (0.34)	b	1.38 (0.01)
	log_10_(*K*_2_)		2.21 (0.09)	3.25 (0.31)	2.86 (0.48)		0.78 (0.01)
	log_10_(β)		5.00^a^	7.81	6.92		2.16^a^
**MT4N**	log_10_(*K*_1_)	b	b	b	2.85 (0.10)	b	1.32 (0.01)
	log_10_(*K*_2_)				2.25 (0.10)		0.72 (0.01)
	log_10_(β)				5.10^a^		2.04^a^

a Noncooperative 1:2 host–guest model.^45^

b Not determined due to poor observed changes or deprotonation.

The global association constants (log_10_(β)) shown in Table 3 evidence that **MT1N** has a higher affinity for H_2_PO_4_^–^ followed by C_6_H_5_CO_2_^–^ and CH_3_CO_2_^–^. The association constants of the complexes between the receptors and oxoanions, excluding bisulfate, can be formed by the combination of conventional and unconventional hydrogen bonding interactions through hydrogens H-9, H-1, H-7, and H-5, especially with H-1 and H-7. On the other hand, the interaction of chloride with the receptors seems to occur mainly through unconventional hydrogen bonds and, to a lesser extent, with hydrogens H-1 and H-7, which may explain the reduced magnitude of the association constants for the complexes with this anion.

Additionally, all systems exhibit a strong decrease in the value of log_10_(*K*_2_), consistent with the idea of noncooperative binding sites, as previously described in the literature.^45−47^ The observed strong penalty for binding of the second anion to the receptor is probably because the anion must break or at least interfere with the intramolecular hydrogen bond. This fact allows us to elucidate the reason why when using the UV–vis technique there was not an adequate response from the chromophores, most likely because the hydrogen closest to these signaling groups participates very little in the recognition process.

Finally, it is important to point out that the differences observed in the magnitudes of the association constants obtained by UV–vis and ^1^H NMR techniques can be attributed to the different concentrations required according to the technique as well as the difference in the physical property being measured and the way the receptors interact with the anions in these systems.^48,49^

### Fluorescence

**Figure 5:**
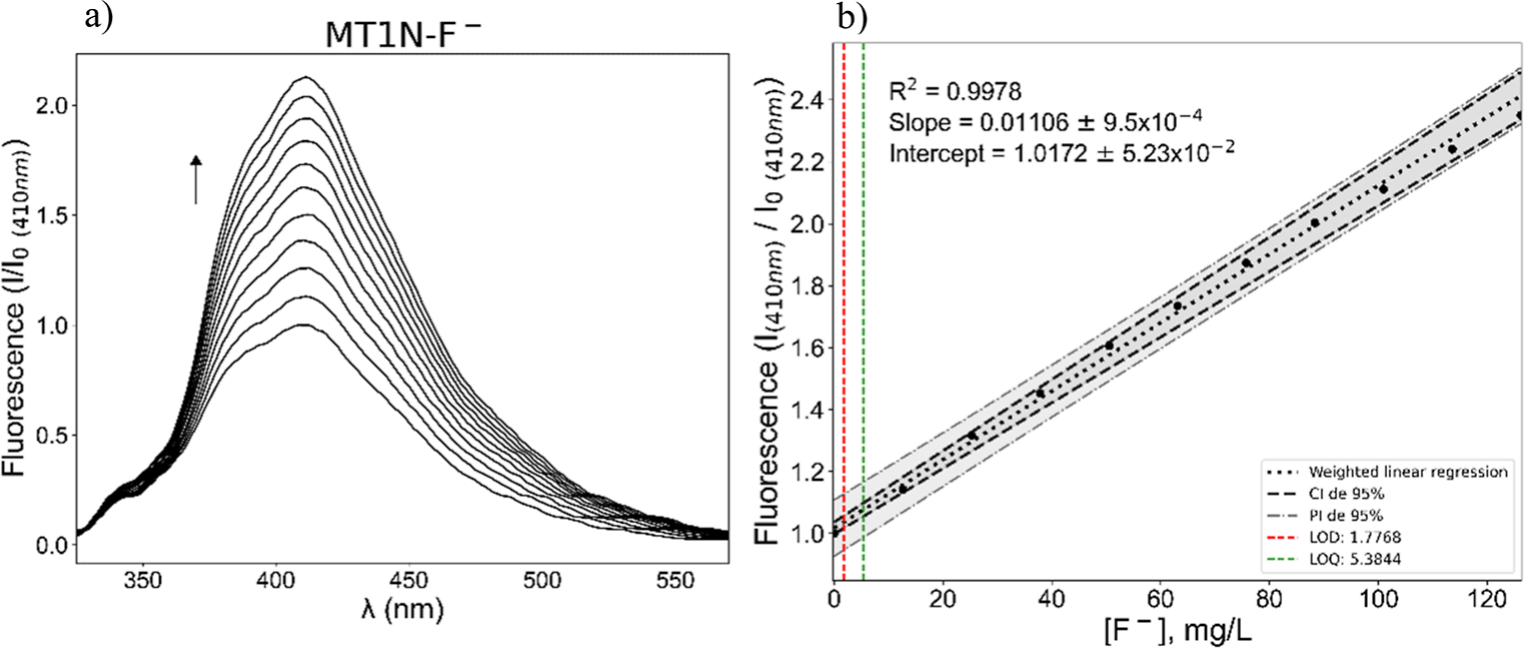
(a) Emission spectra of **MT1N** (1 × 10^–5^ M) in the presence of increasing concentrations of F^–^ (0–4 × 10^–4^ M) in DMSO at 298 K and λ_ex_ = 342 nm. (b) Weighted linear regression of **MT1N**–F^–^.

Considering the structure of **MT1N** and its fluorescent response under UV light in the presence of fluoride, the fluorescence technique was used to evaluate the receptor response to basic anions F^–^, CH_3_CO_2_^–^, and H_2_PO_4_^–^. The emission spectrum of free **MT1N** shows a peak at 410 nm and a shoulder at 376 nm, both bands belonging to the monomeric species of the receptor, which was confirmed through emission measurements at different concentrations (5 × 10^–7^ to 5 × 10^–5^ M), to observe possible aggregation effects and to establish the linear response range of fluorescence emission. From these experiments, calibration curves were generated to study the emission response of **MT1N** in the presence of basic anions. As a representative example, Figure 5 presents the emission spectra of **MT1N** at different fluoride concentrations, while the observed responses for the receptor with CH_3_CO_2_^–^ and H_2_PO_4_^–^ can be seen in Figures S33 and S34, respectively.

Figure 5a shows that the fluorescence intensity increases as the concentration of fluoride increases; this dependency closely approximates linearity. It is important to note that in this study, no experiments were conducted to precisely determine the mechanisms observed for the fluorescent response of **MT1N** in the presence of the anions. However, based on the evidence obtained from UV–vis and ^1^H NMR, as well as previous reports in the literature for similar systems,^31^ it is reasonable to infer that the increase in **MT1N** fluorescence with fluoride is primarily due to the deprotonation of **MT1N** and the subsequent charge delocalization in the rings of the fluorophore group.

On the other hand, considering the systems of **MT1N** with acetate and monobasic phosphate, a likely mechanism is photoinduced proton transfer, which consists of the formation of a tautomer due to the transfer of a proton from the donor species to the anion. From the regression analysis of the data from the experiments (Figure 4b), the limit of detection (LOD) and the LOQ values were obtained, which are summarized in Table 4.

**Table 4:** LOD and LOQ Obtained from Weighted Linear Regression of Data (λ_em_ = 410 nm, λ_ex_ = 342 nm) for **MT1N** and Basic Anions by Fluorescence, in DMSO at 298 K

	**MT1N**–F–	**MT1N**–CH_3_CO_2_^–^	**MT1N**–H_2_PO_4_^–^
slope	0.0111 (9.5 × 10^–^^4^)^a^	0.00865 (4.7 × 10^–^^4^)^a^	0.00409 (2.3 × 10^–^^4^)^a^
intercept	1.0172 (5.23 × 10^–^^2^)^a^	1.0150 (3.86 × 10^–^^2^)^a^	1.0081 (2.11 × 10^–^^2^)^a^
LOD^b^	1.7768 mg/L	1.0428 mg/L	1.2578 mg/L
LOQ^c^	5.3844 mg/L	3.1601 mg/L	3.8114 mg/L
*R*^2^	0.9978	0.9991	0.9989

a The associated error is indicated in parentheses.

b LOD = 3.3 · wrsd/slope.

c LOQ = 10 · wrsd/slope. Where: wrsd = weighted residual standard deviation.

The data from the table demonstrate that the slopes obtained from the regression curves follow the order F^–^ > CH_3_CO_2_^–^ > H_2_PO_4_^–^, indicating that the presence of fluoride induces a greater change in the fluorescence intensity of **MT1N** at lower concentrations. On the other hand, based on the LOQ values, measurement windows with reasonable certainty of linearity are established, with this window being similar for the three analyzed anions, in the following order: H_2_PO_4_^–^ (131.99 mg/L) > F^–^ (120.82 mg/L) > CH_3_CO_2_^–^ (112.42 mg/L). It is important to note, as a limitation of the results, that these regression curves establish ideal conditions, meaning that the anions are measured without interferents. However, after a simple inspection of the data in Table 4, it is evident that if the anions studied were simultaneously in a sample of interest, they could cause interference between them. Despite this, these results establish a starting point for the potential utilization of these receptors in the future where the effects of interferents and the matrix are considered. However, such analysis extends beyond the objectives of the present study.

#### Molecular Modeling

**Figure 6:**
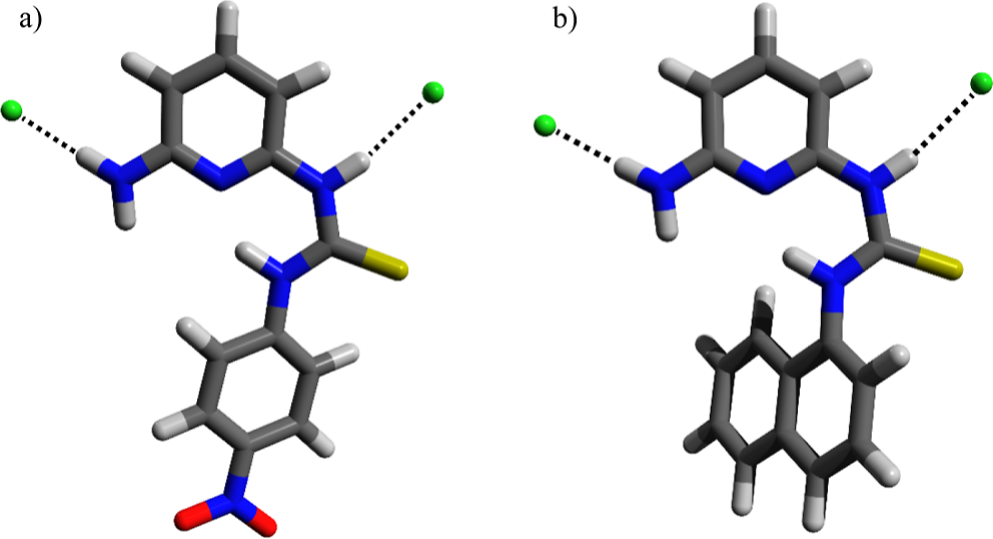
Perspective view of the calculated molecular structures of (a) **MT4N**–Cl^–^ and (b) **MT1N**–Cl^–^ with the B3LYP/6-31G* level of theory in DMSO.

The geometry-optimized molecular structures obtained for the complexes formed between **MT4N** and **MT1N** with the chloride anion, in DMSO with the B3LYP/6-31G* level of theory, are shown in Figure 6. It is important to mention that these models are derived from NOESY experiments conducted on receptor–chloride complexes. As such, they represent systems that rely on hydrogen bonding without undergoing deprotonation (Figures S35 and S36).

According to the calculated molecular structure of the **MT4N**–Cl^–^ 1:2 complex shown in Figure 6a, the receptor interacts with chloride anions through two strong hydrogen bonds; both are indicated in the form of dashed lines. One hydrogen bond is formed between the thiourea unit of the receptor and a chloride, with a N–H···Cl^–^ distance of 2.364 Å and an angle of 168.7°. The other hydrogen bond is formed between one hydrogen from the amine group and the second chloride anion with a N–H···Cl^–^ distance of 2.307 Å and an angle of 179.4°.

On the other hand, Figure 6b shows the molecular structure of the **MT1N**–Cl^–^ 1:2 complex. Like its analogous complex described above, one hydrogen from thiourea is bonded to a chloride with a N–H···Cl^–^ distance of 2.396 Å and an angle of 168.4°. As for the other chloride, it strongly interacts with the amine group with a distance N–H···Cl^–^ of 2.312 Å and an angle of 178.5°. Figure S37 presents the molecular structure of the 1:2 **MT1N**–CH_3_CO_2_^–^ complex. Finally, it is important to mention that these models agree with the results obtained by UV–vis and ^1^H NMR studies.

## Conclusions

The synthesis of a new receptor, **MT1N**, has been reported, along with an improved synthesis method for **MT4N**. Likewise, **MT1N** and **MT4N** were evaluated as chemosensors for anions of diverse geometries and basicity. In this context, **MT4N** can signal the presence of fluoride with colorimetric changes visible to the naked eye, while **MT1N** selectively responds to fluoride under UV light (λ = 265 nm). Molecular recognition studies by UV–vis demonstrate that **MT4N** has a higher affinity for fluoride than for other anions. Additionally, it was observed for systems involving both receptors and basic anions that a greater hydrogen bond strength lessens the probability of deprotonation. On the other hand, studies by ^1^H NMR show that the receptors interact with the anions using two sites: the amino group and the thiourea group. The association constants obtained by ^1^H NMR indicate that **MT1N** interacts strongly with almost all of the anions studied in this work, especially with H_2_PO_4_^–^. Similarly, using this technique, it was observed that **MT1N** is less susceptible to deprotonation than **MT4N** due to the differences in their acidities. **MT1N** exhibited fluorescence enhancement in the presence of F^–^, CH_3_CO_2_^–^, and H_2_PO_4_^–^, although it does not exhibit selectivity for any of the previously mentioned anions by this technique. We are currently working on coordination studies with these receptors and similar compounds, as well as on the synthesis of new analog receptors and their solid phase support focused on the recognition and detection of anions, cations, and salts.

## Supporting Information Available

^1^H, ^13^C NMR, and FT-IR spectra of MT4N and MT1N, colorimetric response of **MT4N** and **MT1N** with basic anions (F^–^, CH_3_CO_2_^–^, and H_2_PO_4_^–^), UV–vis, ^1^H NMR titrations, and 2D NOESY for complexes between the receptors (**MT4N** and **MT1N**) and various salts, weighted linear regression for **MT1N** with CH_3_CO_2_^–^ and H_2_PO_4_^–^ by fluorescence in DMSO at 298 K, and molecular modeling of the **MT1N**–acetate complex (PDF)
