# Supplementary material for: Thiourea-Based Receptors for Anion Recognition and Signaling
Source: ACS Omega. 2024 Jan 17;9(4):4412–22. doi: 10.1021/acsomega.3c06861 (PMC10832000; doi:10.1021/acsomega.3c06861)
Supplement: Supplementary file 1 — ao3c06861_si_001.pdf [file ao3c06861_si_001.pdf]

Supporting Information

Thiourea-based receptors for anion recognition and signaling

Jancarlo Gomez-Vega,[1] Adrian Vasquez-Cornejo,[1] Octavio Juárez-Sánchez,[2] David O. Corona-Martínez,[3] Adrián Ochoa-Terán,[4] Karla A. López-Gastelum,[5] Rogerio R. Sotelo-Mundo,[5] Hisila Santacruz-Ortega,[1] Juan Carlos Gálvez-Ruiz,[3] Refugio Pérez-González,[1] and Karen Ochoa Lara[1*]

[1] Departamento de Investigación en Polímeros y Materiales, Universidad de Sonora, Rosales y Encinas s/n, Col. Centro CP 83000. Hermosillo, Sonora, México. *Corresponding author. E-mail: karen.ochoa@unison.mx

[2] Departamento de Investigación en Física, Universidad de Sonora, Rosales y Encinas s/n, Col. Centro CP 83000. Hermosillo, Sonora, México.

[3] Departamento de Ciencias Químico Biológicas, Universidad de Sonora, Rosales y Encinas s/n, Col. Centro CP 83000. Hermosillo, Sonora, México.

[4] Centro de Graduados e Investigación en Química, Instituto Tecnológico de Tijuana, Blvd. Industrial S/N CP 22510. Tijuana, Baja California, México.

[5] Laboratorio de Estructura Biomolecular. Centro de Investigación en Alimentación y Desarrollo, A. C., Gustavo Enrique Astiazaran Rosas, No. 46. CP 83304. Hermosillo, Sonora, México.

Table of contents

Figure S1. ^1H NMR spectrum of **MT4N** in DMSO-d_6 at 298 K. 5
Figure S2. ^{13}C NMR spectrum of **MT4N** in DMSO-d_6 at 298 K. 5
Figure S3. ^1H NMR spectrum of **MT1N** in DMSO-d_6 at 298 K. 6
Figure S4. ^{13}C NMR spectrum of **MT1N** in DMSO-d_6 at 298 K. 6
Figure S5. FTIR-ATR spectrum of **MT4N**. 7
Figure S6. FTIR-ATR spectrum of **MT1N**. 7
Figure S7. The 2D NOESY spectrum of **MT4N** in DMSO-d_6. 8
Figure S8. The 2D NOESY spectrum of **MT1N** in DMSO-d_6. 8
Figure S9. Absorption spectra of **MT4N** at several concentrations (1-8x10^{-5} M). The inset is a linear regression at 355 nm to determine the molar extinction coefficient. 9
Figure S10. Absorption spectra of **MT1N** at several concentrations (1-8x10^{-5} M). The inset is a linear regression at 331 nm to determine the molar extinction coefficient. 9
Figure S11. a) Colorimetric response of **[MT4N] =** 3 x10^{-5} M with 6 equivalents of anions. b) **[MT1N] =** 6 x10^{-5} M with 12 equivalents of anions. c) **[MT1N] =** 6 x10^{-5} M with 12 equivalents of anions under UV light (λ = 365 nm). All anions were used in their TBA salts forms. 10
Figure S12. Absorption spectra of **MT1N** (3×10^{-5} M) in the presence of increasing concentrations of Cl^- (0-4.39×10^{-3} M) in DMSO at 298 K. The upper right inset shows the relative abundance of the different species during the titration, where H = receptor and G = anion guest. The dashed lines represent the theoretical profiles obtained from data fitting. 11
Figure S13. Absorption spectra of **MT1N** (3×10^{-5} M) in the presence of increasing concentrations of NO_3^- (0-4.72×10^{-3} M) in DMSO at 298 K. The upper right inset shows the relative abundance of the different species during the titration, where H = receptor and G = anion guest. The dashed lines represent the theoretical profiles obtained from data fitting. 11
Figure S14. Absorption spectra of **MT1N** (3×10^{-5} M) in the presence of increasing concentrations of HSO_4^- (0-5.91×10^{-3} M) in DMSO at 298 K. The upper right inset shows the relative abundance of the different species during the titration, where H = receptor and G = anion guest. The dashed lines represent the theoretical profiles obtained from data fitting. 12
Figure S15. Absorption spectra of **MT1N** (3×10^{-5} M) in the presence of increasing concentrations of $H_2PO_4^-$ (0-1.9×10^{-3} M) in DMSO at 298 K. The upper right inset shows the relative abundance of the different species during the titration, where H = receptor and G = anion guest. The dashed lines represent the theoretical profiles obtained from data fitting. (Non-reproducible experiment). 12
Figure S16. Absorption spectra of **MT1N** (3×10^{-5} M) in the presence of increasing concentrations of $CH_3CO_2^-$ (0-2.19×10^{-3} M) in DMSO at 298 K. The upper right inset shows the relative abundance of the different species during the titration, where H = receptor. The dashed lines represent the theoretical profiles obtained from data fitting. 13
Figure S17. Absorption spectra of **MT4N** (3.6×10^{-5} M) in the presence of increasing concentrations of Cl^- (0-5.25×10^{-3} M) in DMSO at 298 K. The upper right inset shows the relative abundance of the different species during the titration, where H = receptor and G = anion guest. The dashed lines represent the theoretical profiles obtained from data fitting. 13

Figure S18. Absorption spectra of **MT4N** (3.6×10^{-5} M) in the presence of increasing concentrations of NO_3^- ($0\text{-}3.52\times10^{-2}$ M) in DMSO at 298 K. The upper right inset shows the relative abundance of the different species during the titration, where H = receptor and G = anion guest. The dashed lines represent the theoretical profiles obtained from data fitting. 14
Figure S19. Absorption spectra of **MT4N** (3.6×10^{-5} M) in the presence of increasing concentrations of HSO_4^- ($0\text{-}9.55\times10^{-3}$ M) in DMSO at 298 K. 14
Figure S20. Absorption spectra of **MT4N** (3.6×10^{-5} M) in the presence of increasing concentrations of $H_2PO_4^-$ ($0\text{-}1.36\times10^{-3}$ M) in DMSO at 298 K. The upper right inset shows the relative abundance of the different species during the titration, where H = receptor. The dashed lines represent the theoretical profiles obtained from data fitting. 15
Figure S21. Absorption spectra of **MT4N** (3.6×10^{-5} M) in the presence of increasing concentrations of $CH_3CO_2^-$ ($0\text{-}3.88\times10^{-4}$ M) in DMSO at 298 K. The upper right inset shows the relative abundance of the different species during the titration, where H = receptor and G = anion guest. The dashed lines represent the theoretical profiles obtained from data fitting. 15
Figure S22. ^{1}H NMR spectra of **MT4N** (3 mM) with increasing concentration of $CH_3CO_2^-$ ($0\text{-}6x10^{-3}$ M) in DMSO-d_6 at 298 K. 16
Figure S23. ^{1}H NMR spectra of **MT4N** (3 mM) with increasing concentration of $H_2PO_4^-$($0\text{-}6x10^{-3}$ M) in DMSO-d_6 at 298 K. 16
Figure S24. a) Selected spectra from the titration of **MT4N** (3 mM) with $C_6H_5CO_2^-$ ($0\text{-}1.20x10^{-4}$ M) in DMSO-d_6 at 298 K. b) Theoretical fit of experimentally measured chemical shift using a 1:1 model by least squares regression. c) Abundance of the different species during the titration, where H = receptor and G = anion guest. 17
Figure S25. a) Selected spectra from the titration of **MT4N** (3 mM) with Cl^- (0-0.06 M) in DMSO-d_6 at 298 K. b) Theoretical fit of experimentally measured chemical shift using a 1:2 model by least squares regression. c) Abundance of the different species during the titration, where H = receptor and G = anion guest. 18
Figure S26. ^{1}H NMR spectra of **MT4N** (3 mM) with increasing concentration of HSO_4^-($0\text{-}2.10x10^{-4}$ M) in DMSO-d_6 at 298 K. 19
Figure S27. ^{1}H NMR spectra of **MT1N** (3 mM) with increasing concentration of F^- ($0\text{-}9x10^{-3}$ M) in DMSO-d_6 at 298 K. 20
Figure S28. a) Selected spectra from the titration of **MT1N** (3 mM) with $C_6H_5CO_2^-$ ($0\text{-}2.25x10^{-4}$ M) in DMSO-d_6 at 298 K. b) Theoretical fit of experimentally measured chemical shift using a 1:2 model by least squares regression. c) Abundance of the different species during the titration, where H = receptor and G = anion guest. 21
Figure S29. a) Selected spectra from the titration of **MT1N** (3 mM) with $CH_3CO_2^-$ ($0\text{-}12.51x10^{-3}$ M) in DMSO-d_6 at 298 K. b) Theoretical fit of experimentally measured chemical shift using a 1:2 model by least squares regression. c) Abundance of the different species during the titration, where H = receptor. 22
Figure S30. a) Selected spectra from the titration of **MT1N** (3 mM) with $H_2PO_4^-$ ($0\text{-}2.40x10^{-4}$ M) in DMSO-d_6 at 298 K. b) Theoretical fit of experimentally measured chemical shift using a 1:2 model by least squares regression. c) Abundance of the different species during the titration, where H = receptor and G = anion guest. 23

Figure S31. Selected spectra from the titration of **MT1N** (3 mM) with HSO_4^- (0-2.25x10^{-4} M) in DMSO-d_6 at 298 K. 24
Figure S32. a) Selected spectra from the titration of **MT1N** (3 mM) with Cl^- (0-0.06 M) in DMSO-d_6 at 298 K. b) Theoretical fit of experimentally measured chemical shift using a 1:2 model by least squares regression. c) Abundance of the different species during the titration, where H = receptor and G = anion guest. 25
Figure S33. Emission spectra of **MT1N** (1×10^{-5} M) in the presence of increasing concentrations of $CH_3CO_2^-$ (0-4×10^{-4} M) in DMSO at 298 K. b) Weighted linear regression of **MT1N**-$CH_3CO_2^-$ λ_{ex} = 342 nm. 26
Figure S34. Emission spectra of **MT1N** (1×10^{-5} M) in the presence of increasing concentrations of $H_2PO_4^-$ (0-4×10^{-4} M) in DMSO at 298 K. b) Weighted linear regression of **MT1N**-$H_2PO_4^-$. λ_{ex} = 342 nm. 26
Figure S35. NOESY spectrum of [**MT4N**] = 3mM with 20 equivalents of TBAC in DMSO-d_6 at 289 K 27
Figure S36. NOESY spectrum of [**MT1N**] = 3mM with 20 equivalents of TBAC in DMSO-d_6 at 289 K 27
Figure S37. A perspective view of the calculated molecular structure of **MT1N**−$CH_3CO_2^-$ with the B3LYP/6-31G* level of theory, in DMSO. 28

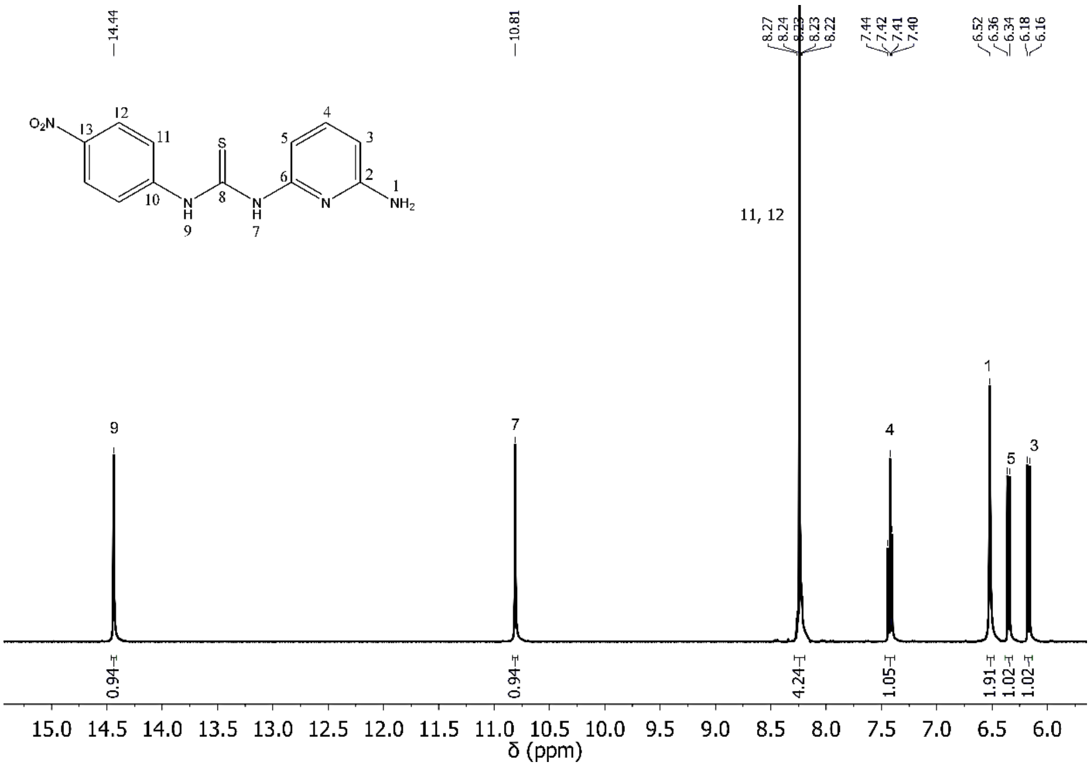

Figure S1. ^{1}H NMR spectrum of **MT4N** in DMSO-d_6 at 298 K.

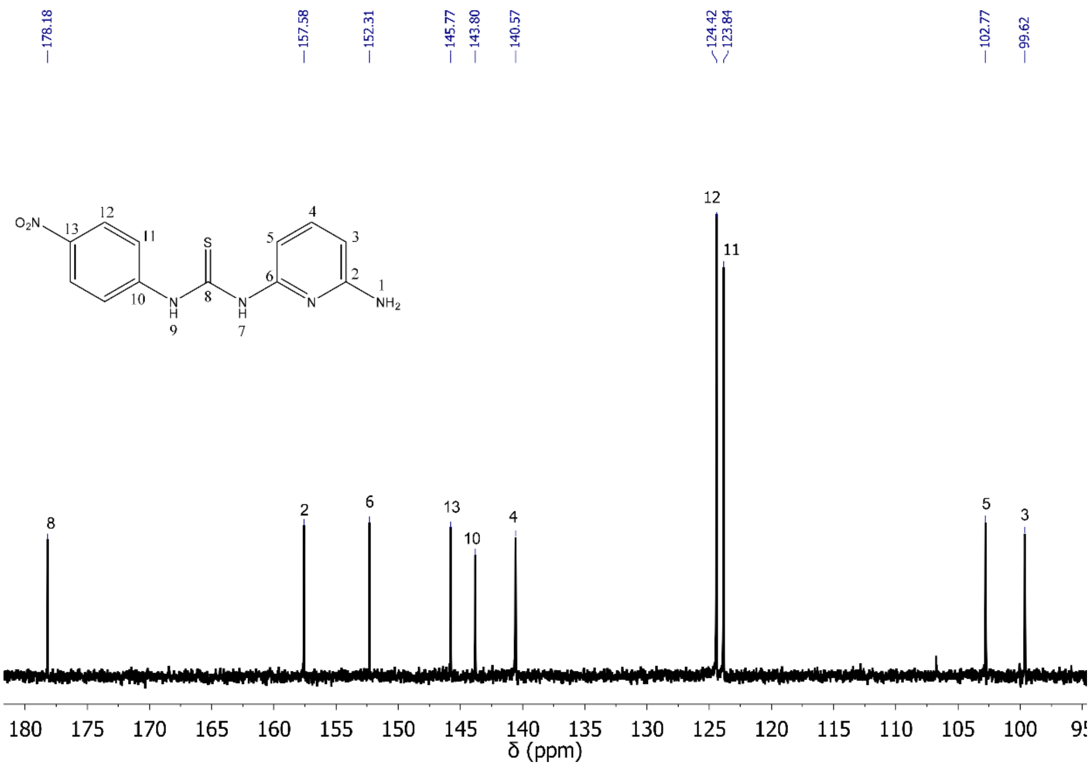

Figure S2. ^{13}C NMR spectrum of **MT4N** in DMSO-d_6 at 298 K.

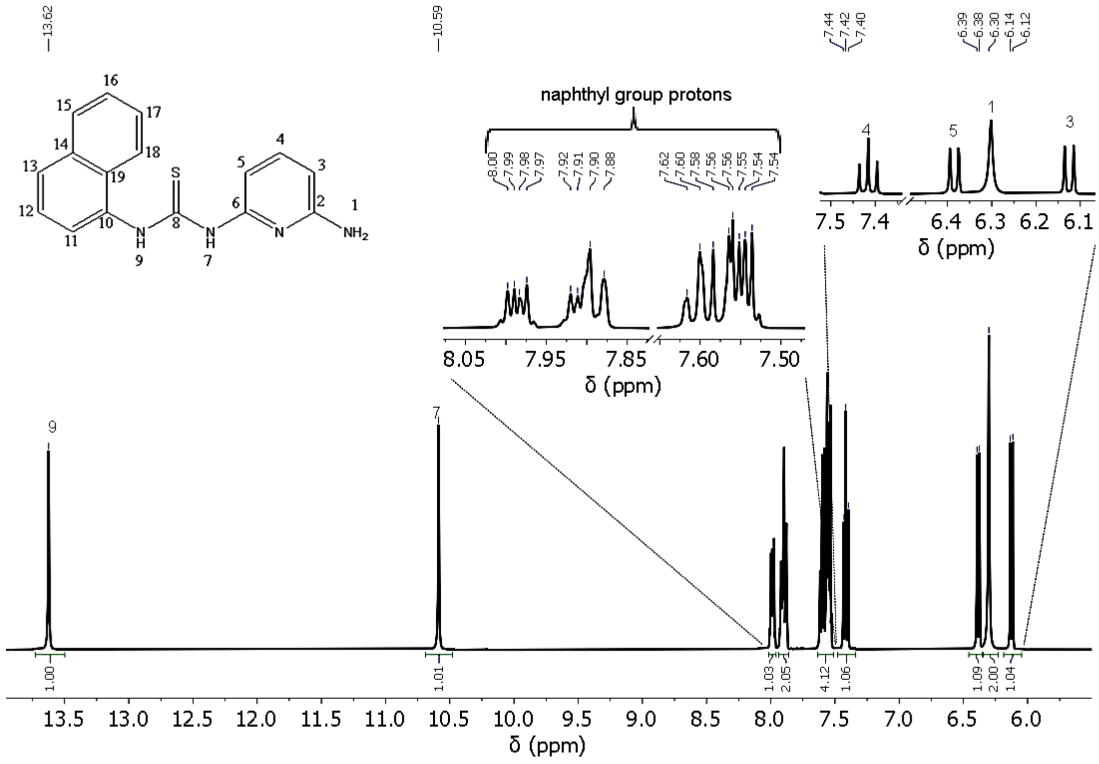

Figure S3. ^{1}H NMR spectrum of **MT1N** in DMSO-d_6 at 298 K.

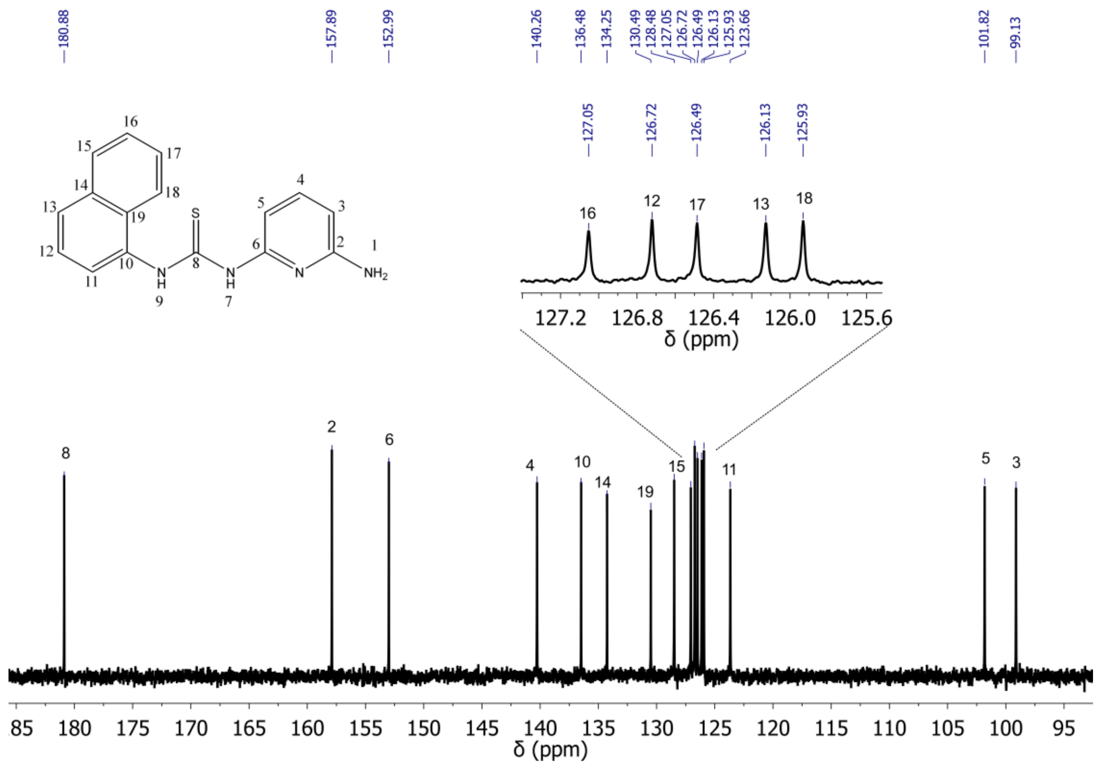

Figure S4. ^{13}C NMR spectrum of **MT1N** in DMSO-d_6 at 298 K.

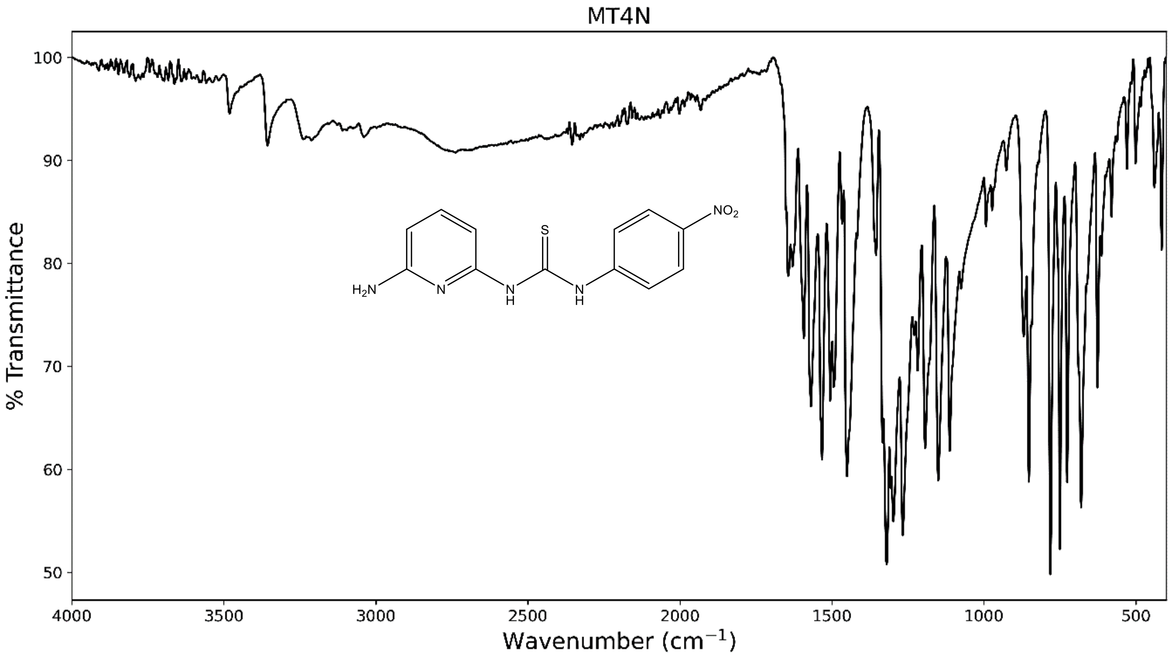

Figure S5. FTIR-ATR spectrum of **MT4N**.

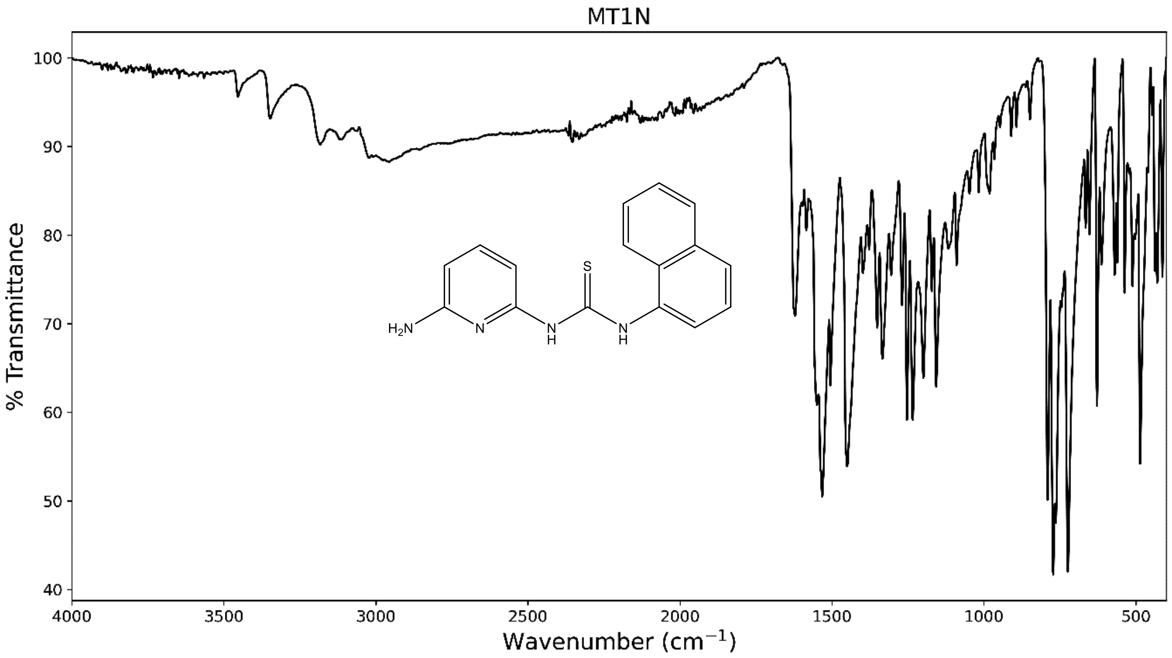

Figure S6. FTIR-ATR spectrum of **MT1N**.

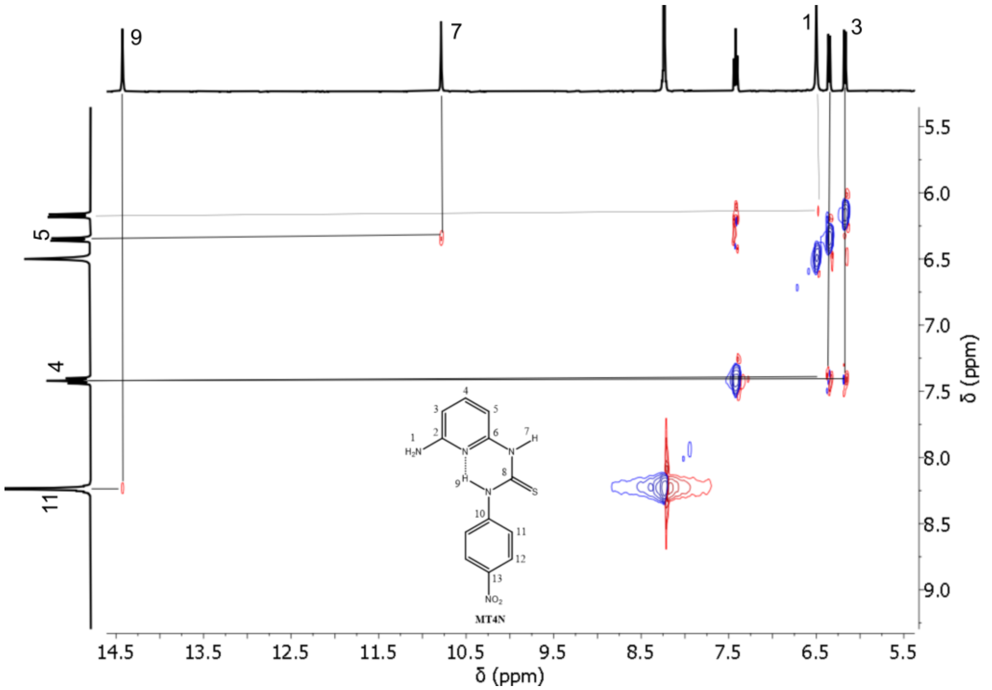

Figure S7. The 2D NOESY spectrum of **MT4N** in DMSO-d_6.

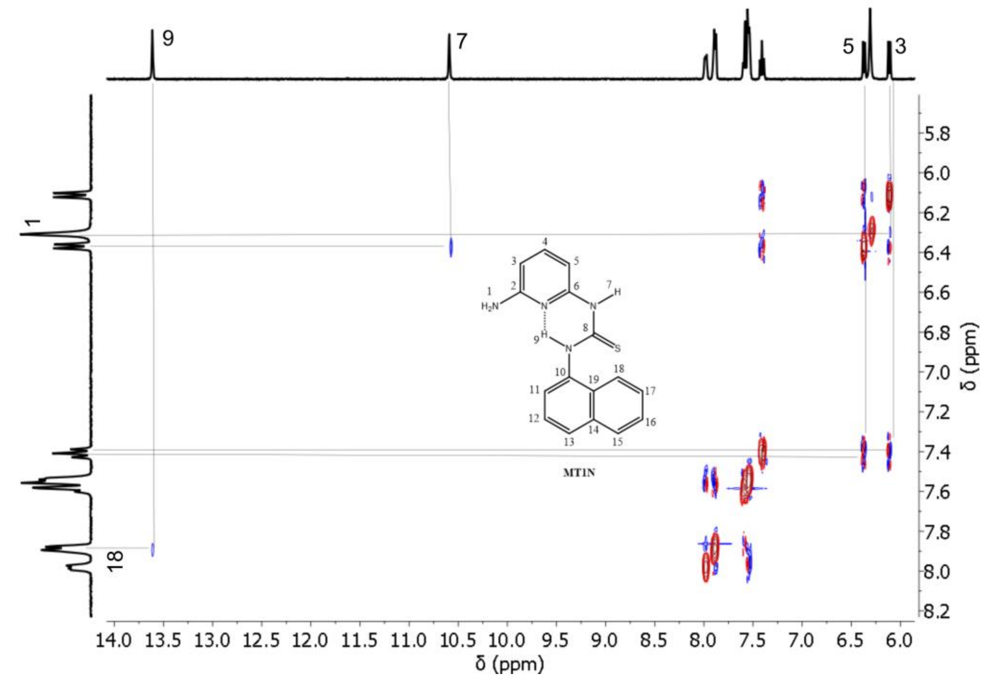

Figure S8. The 2D NOESY spectrum of **MT1N** in DMSO-d_6.

UV-Vis

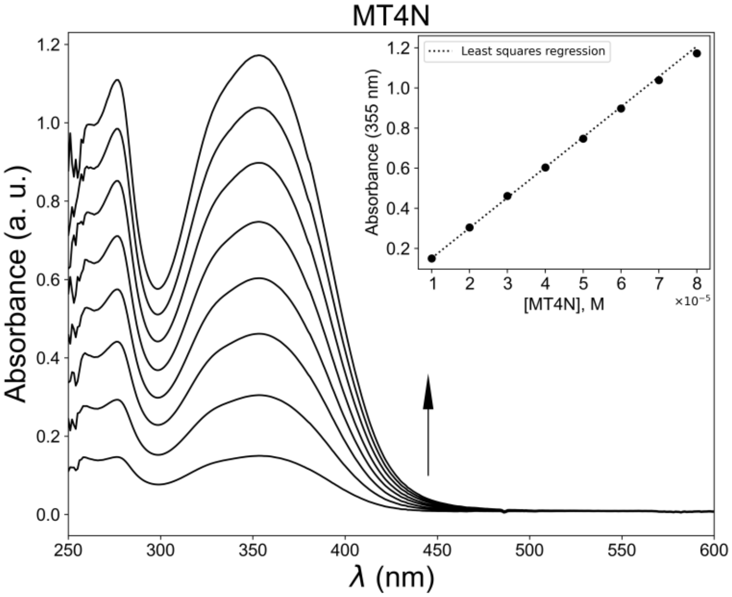

Figure S9. Absorption spectra of **MT4N** at several concentrations (1-8x10^{-5} M). The inset is a linear regression at 355 nm to determine the molar extinction coefficient.

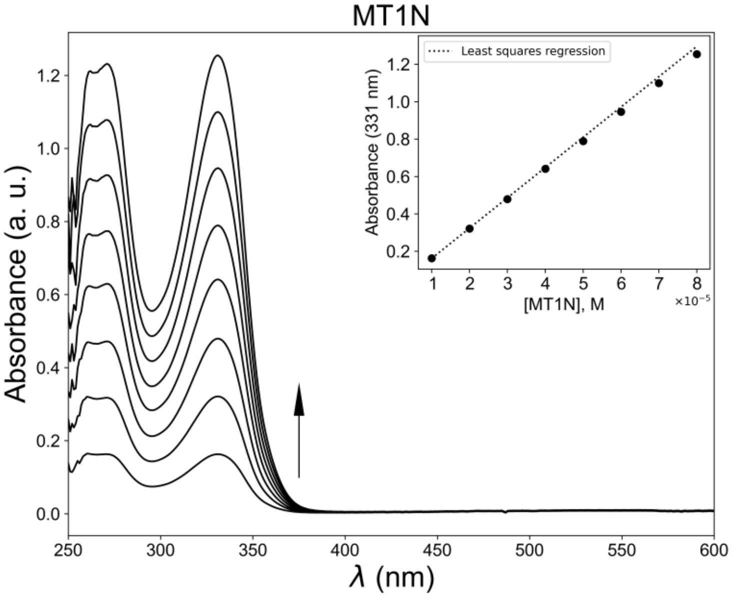

Figure S10. Absorption spectra of **MT1N** at several concentrations (1-8x10^{-5} M). The inset is a linear regression at 331 nm to determine the molar extinction coefficient.

a)

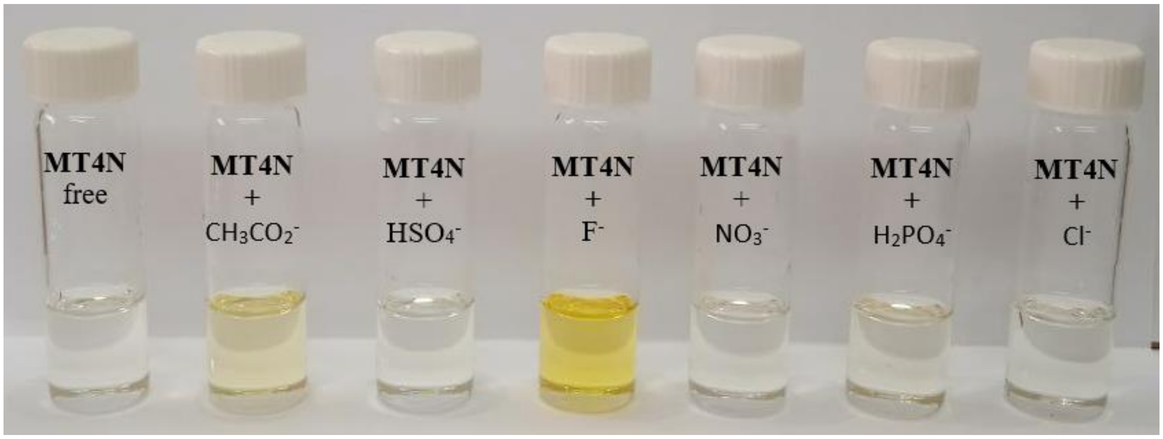

b)

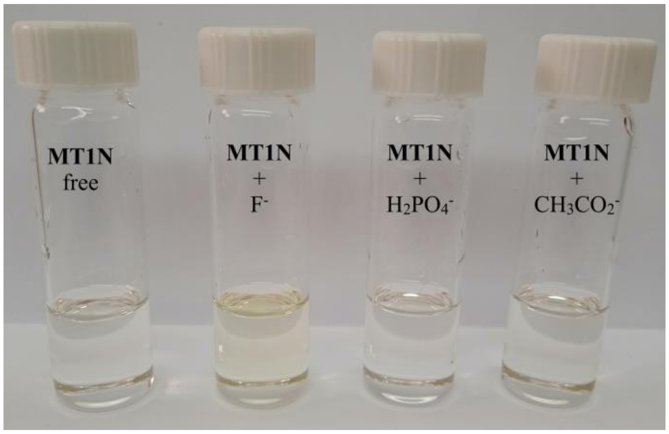

c)

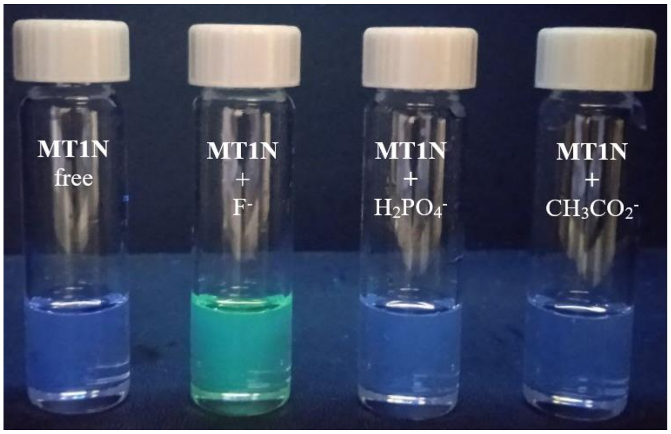

Figure S11. a) Colorimetric response of [**MT4N**] **=** 3 x10^{-5} M with 6 equivalents of anions. b) [**MT1N**] = 6 x10^{-5} M with 12 equivalents of anions. c) [**MT1N**] **=** 6 x10^{-5} M with 12 equivalents of anions under UV light (λ = 365 nm). All anions were used in their TBA salts forms.

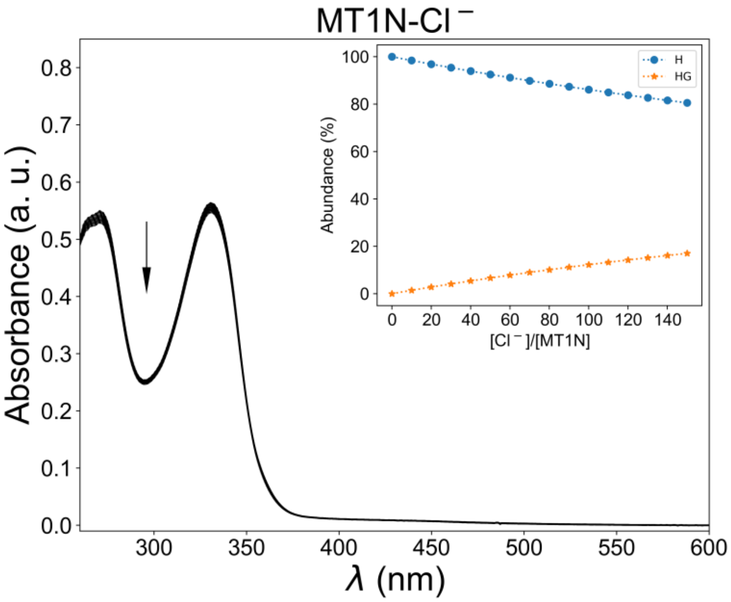

Figure S12. Absorption spectra of **MT1N** (3×10^{-5} M) in the presence of increasing concentrations of Cl^- ($0\text{-}4.39\times10^{-3}$ M) in DMSO at 298 K. The upper right inset shows the relative abundance of the different species during the titration, where H = receptor and G = anion guest. The dashed lines represent the theoretical profiles obtained from data fitting.

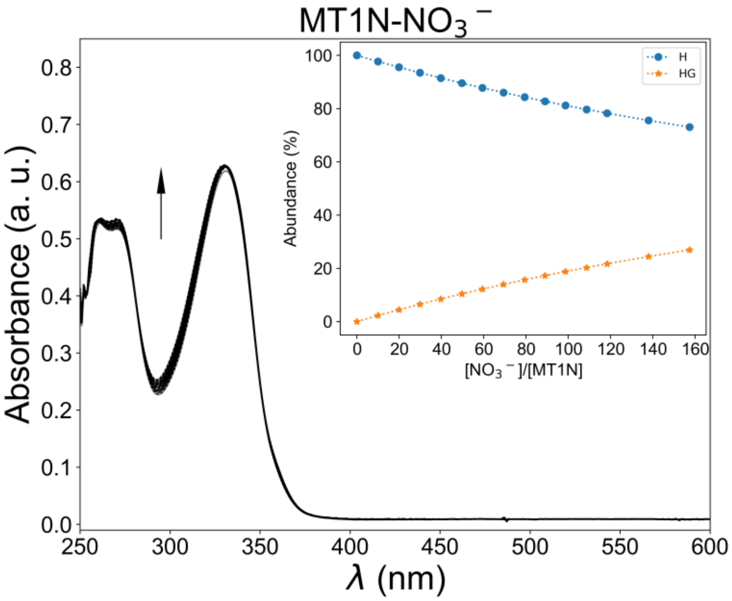

Figure S13. Absorption spectra of **MT1N** (3×10^{-5} M) in the presence of increasing concentrations of NO_3^- ($0\text{-}4.72\times10^{-3}$ M) in DMSO at 298 K. The upper right inset shows the relative abundance of the different species during the titration, where H = receptor and G = anion guest. The dashed lines represent the theoretical profiles obtained from data fitting.

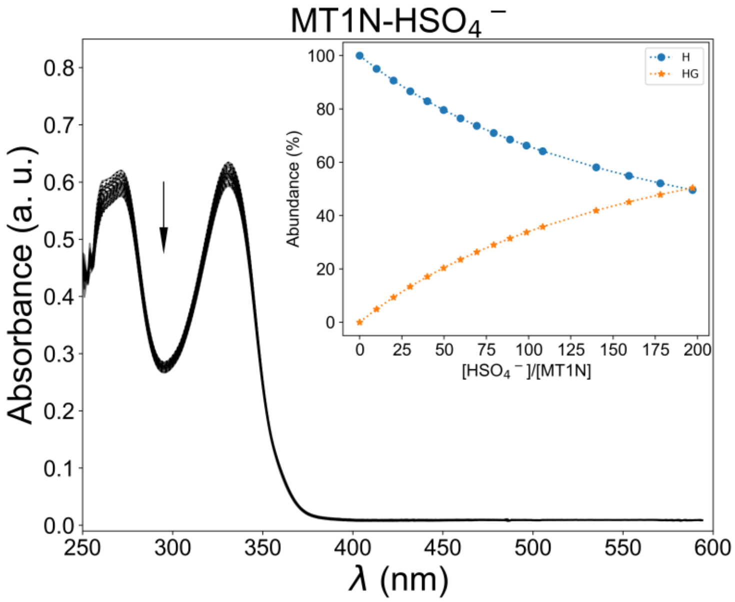

Figure S14. Absorption spectra of **MT1N** (3×10^{-5} M) in the presence of increasing concentrations of HSO_4^- ($0\text{-}5.91\times10^{-3}$ M) in DMSO at 298 K. The upper right inset shows the relative abundance of the different species during the titration, where H = receptor and G = anion guest. The dashed lines represent the theoretical profiles obtained from data fitting.

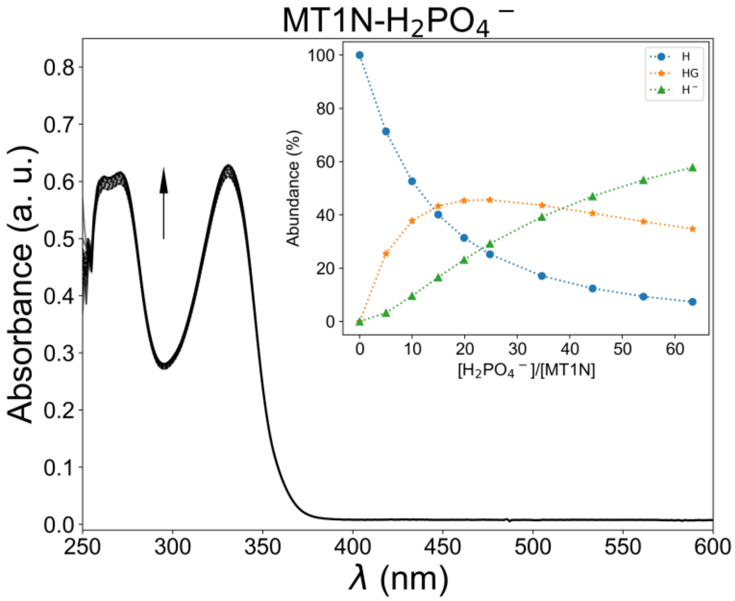

Figure S15. Absorption spectra of **MT1N** (3×10^{-5} M) in the presence of increasing concentrations of $H_2PO_4^-$ ($0\text{-}1.9\times10^{-3}$ M) in DMSO at 298 K. The upper right inset shows the relative abundance of the different species during the titration, where H = receptor and G = anion guest. The dashed lines represent the theoretical profiles obtained from data fitting. (Non-reproducible experiment).

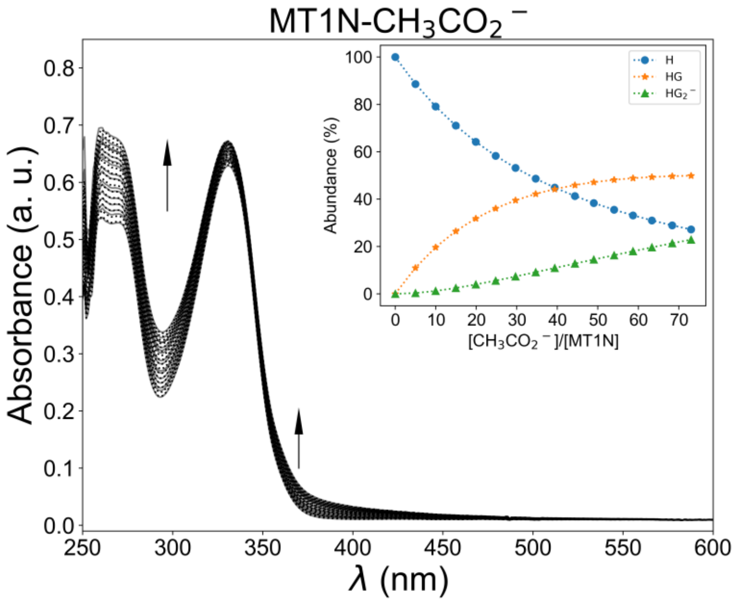

Figure S16. Absorption spectra of **MT1N** (3×10^{-5} M) in the presence of increasing concentrations of $CH_3CO_2^-$ ($0\text{-}2.19\times10^{-3}$ M) in DMSO at 298 K. The upper right inset shows the relative abundance of the different species during the titration, where H = receptor. The dashed lines represent the theoretical profiles obtained from data fitting.

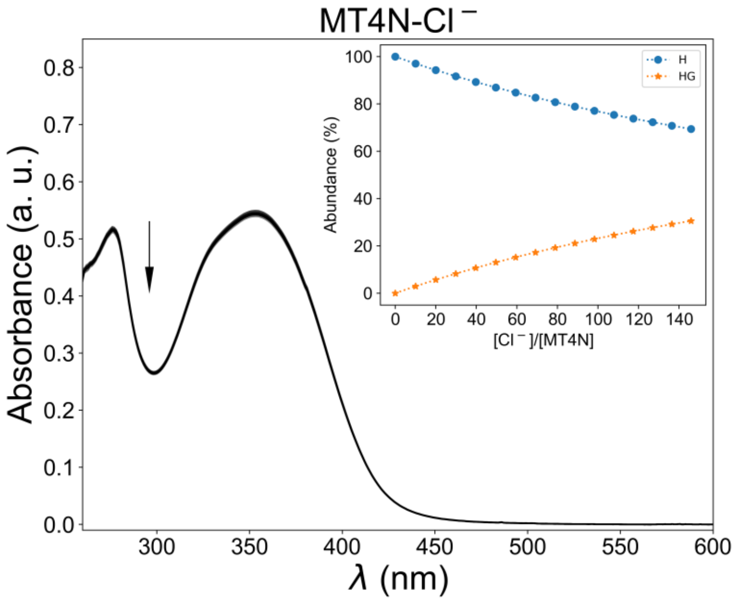

Figure S17. Absorption spectra of **MT4N** (3.6×10^{-5} M) in the presence of increasing concentrations of Cl^- ($0\text{-}5.25\times10^{-3}$ M) in DMSO at 298 K. The upper right inset shows the relative abundance of the different species during the titration, where H = receptor and G = anion guest. The dashed lines represent the theoretical profiles obtained from data fitting.

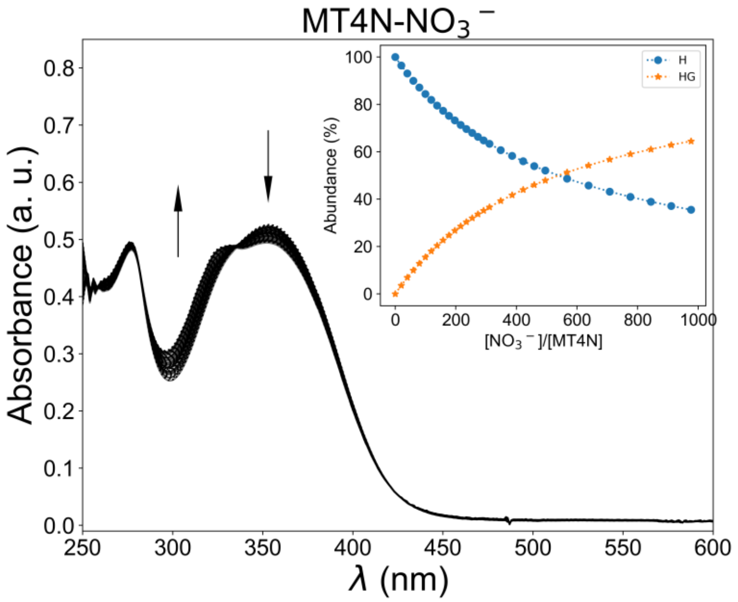

Figure S18. Absorption spectra of **MT4N** (3.6×10^{-5} M) in the presence of increasing concentrations of NO_3^- ($0\text{-}3.52\times10^{-2}$ M) in DMSO at 298 K. The upper right inset shows the relative abundance of the different species during the titration, where H = receptor and G = anion guest. The dashed lines represent the theoretical profiles obtained from data fitting.

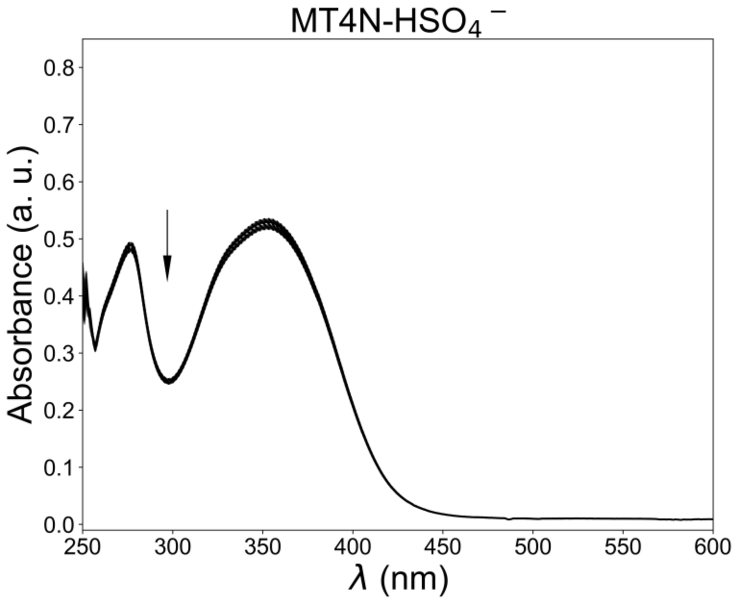

Figure S19. Absorption spectra of **MT4N** (3.6×10^{-5} M) in the presence of increasing concentrations of HSO_4^- ($0\text{-}9.55\times10^{-3}$ M) in DMSO at 298 K.

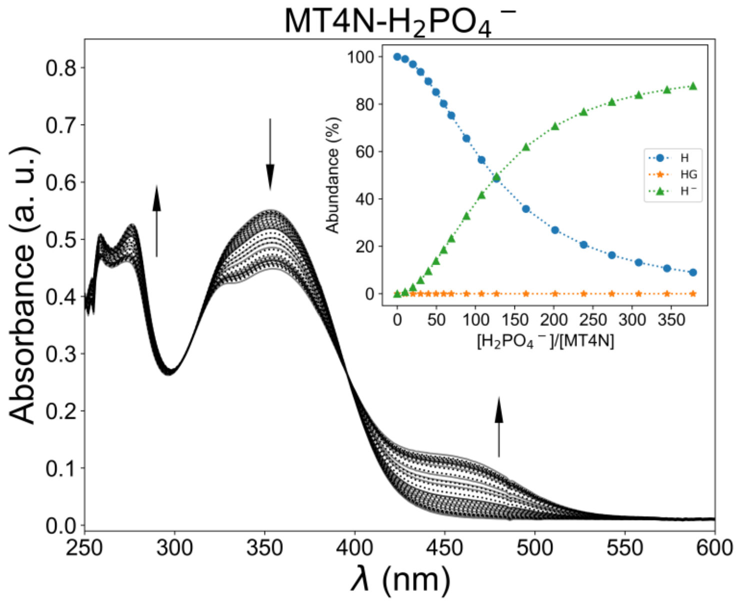

Figure S20. Absorption spectra of **MT4N** (3.6×10^{-5} M) in the presence of increasing concentrations of $H_2PO_4^-$ ($0\text{-}1.36\times10^{-3}$ M) in DMSO at 298 K. The upper right inset shows the relative abundance of the different species during the titration, where H = receptor. The dashed lines represent the theoretical profiles obtained from data fitting.

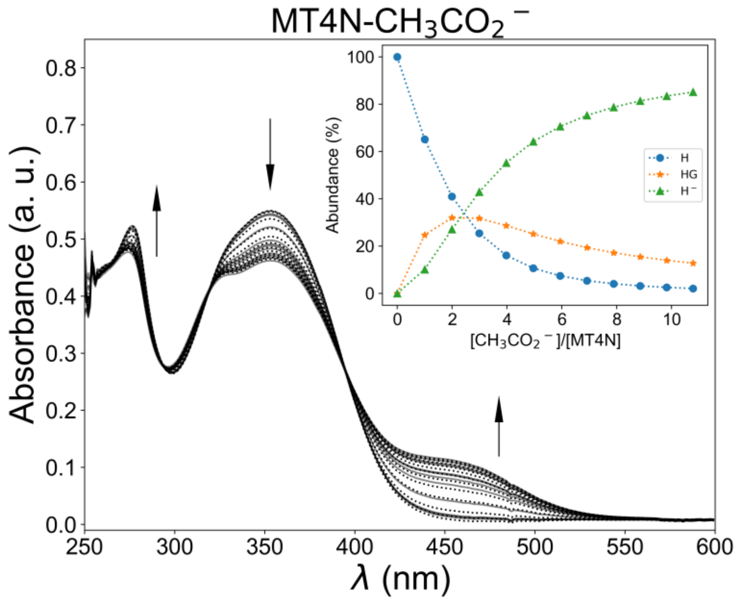

Figure S21. Absorption spectra of **MT4N** (3.6×10^{-5} M) in the presence of increasing concentrations of $CH_3CO_2^-$ ($0\text{-}3.88\times10^{-4}$ M) in DMSO at 298 K. The upper right inset shows the relative abundance of the different species during the titration, where H = receptor and G = anion guest. The dashed lines represent the theoretical profiles obtained from data fitting.

Titrations of NMR ^{1}H

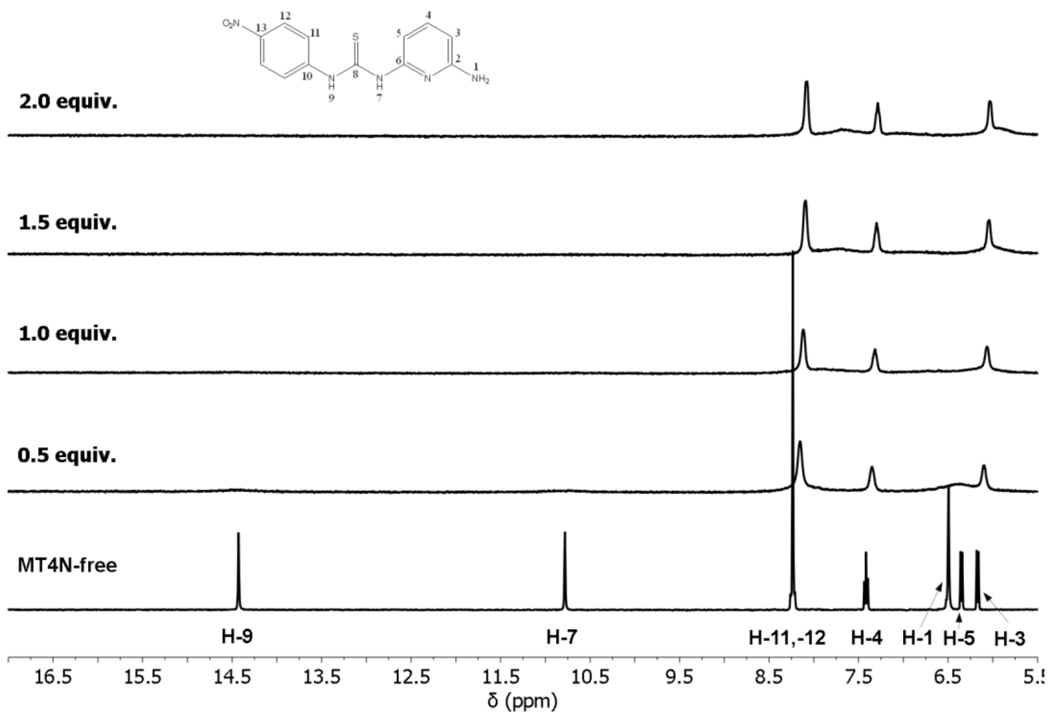

Figure S22. ^{1}H NMR spectra of **MT4N** (3 mM) with increasing concentration of $CH_3CO_2^-$ (0-6x10^{-3} M) in DMSO-d_6 at 298 K.

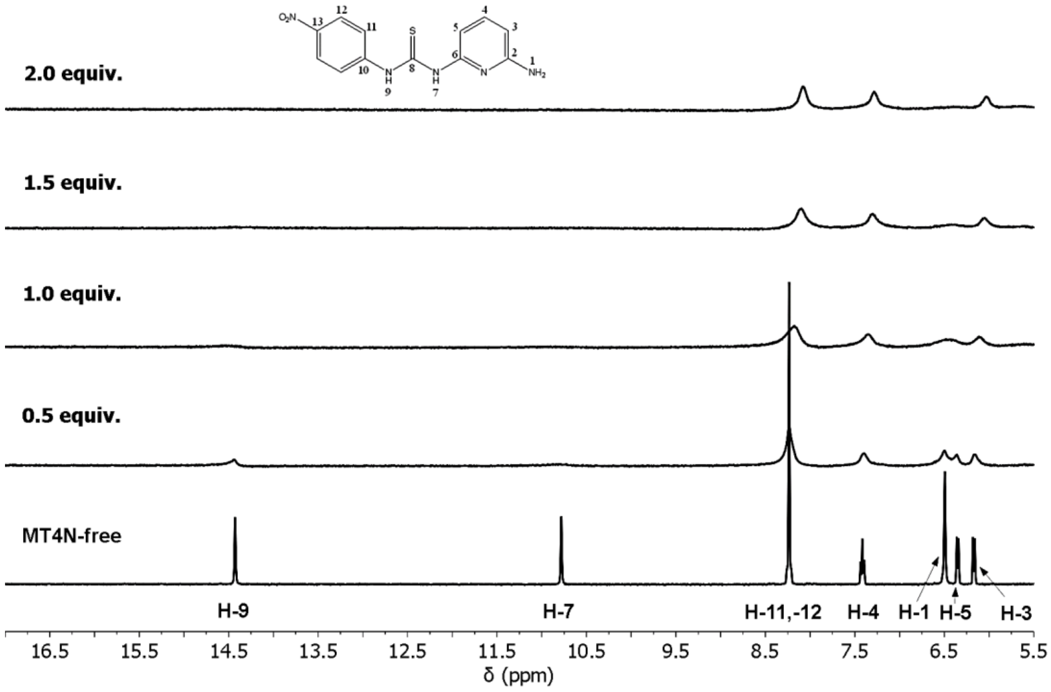

Figure S23. ^{1}H NMR spectra of **MT4N** (3 mM) with increasing concentration of $H_2PO_4^-$(0-6x10^{-3} M) in DMSO-d_6 at 298 K.

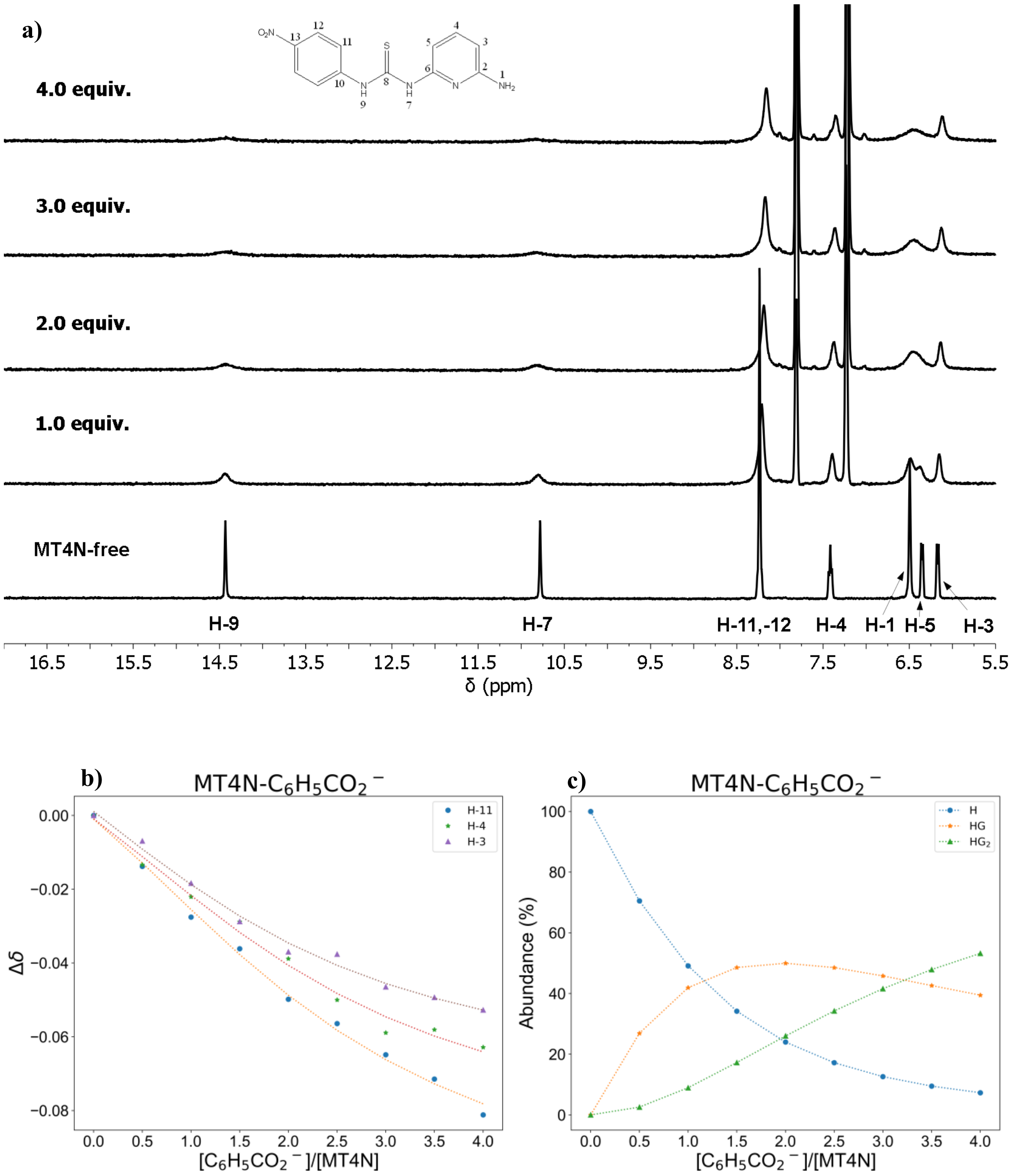

Figure S24. a) Selected spectra from the titration of **MT4N** (3 mM) with $C_6H_5CO_2^-$ (0-1.20×10^{-4} M) in DMSO-d_6 at 298 K. b) Theoretical fit of experimentally measured chemical shift using a 1:1 model by least squares regression. c) Abundance of the different species during the titration, where H = receptor and G = anion guest.

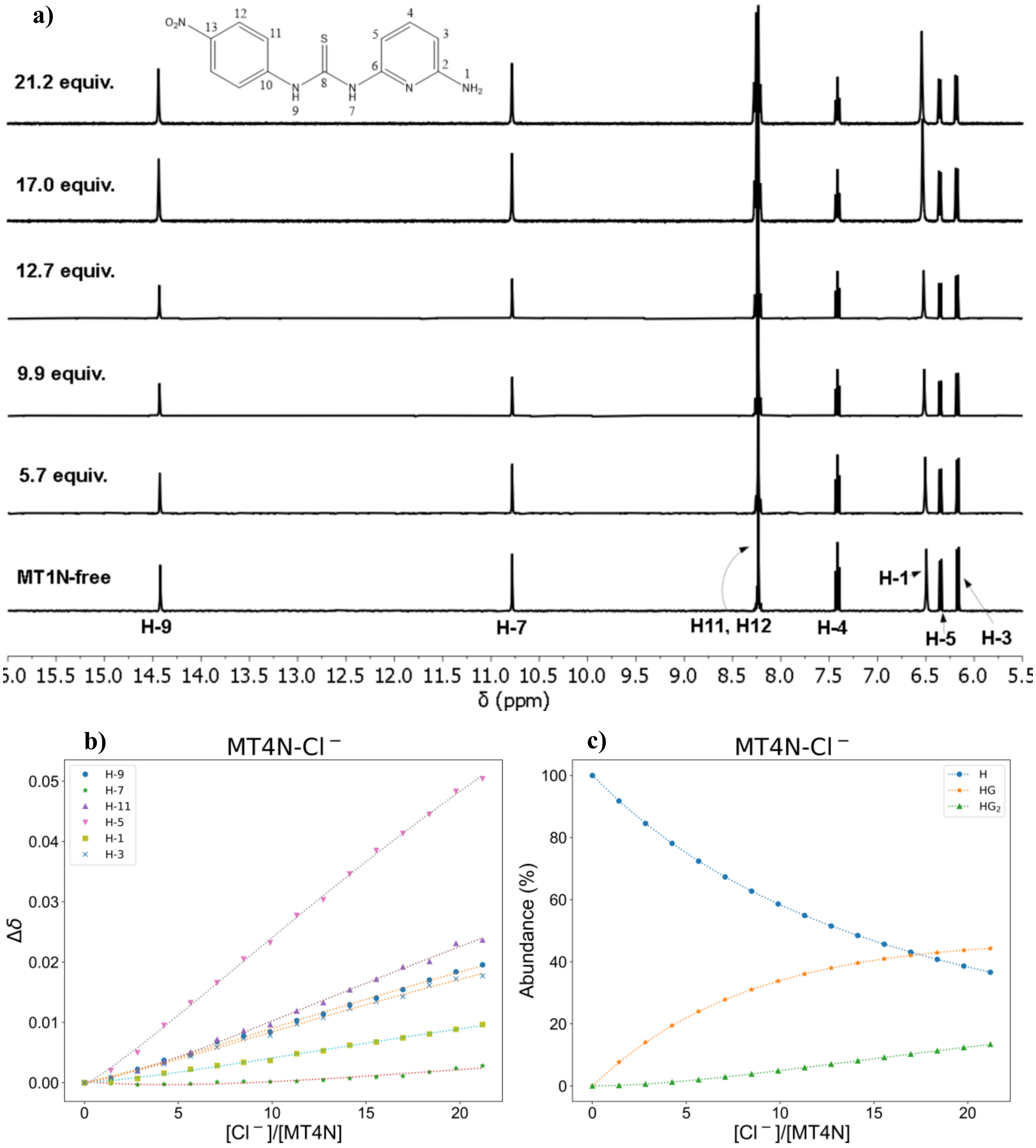

Figure S25. a) Selected spectra from the titration of **MT4N** (3 mM) with Cl^- (0-0.06 M) in DMSO-d_6 at 298 K. b) Theoretical fit of experimentally measured chemical shift using a 1:2 model by least squares regression. c) Abundance of the different species during the titration, where H = receptor and G = anion guest.

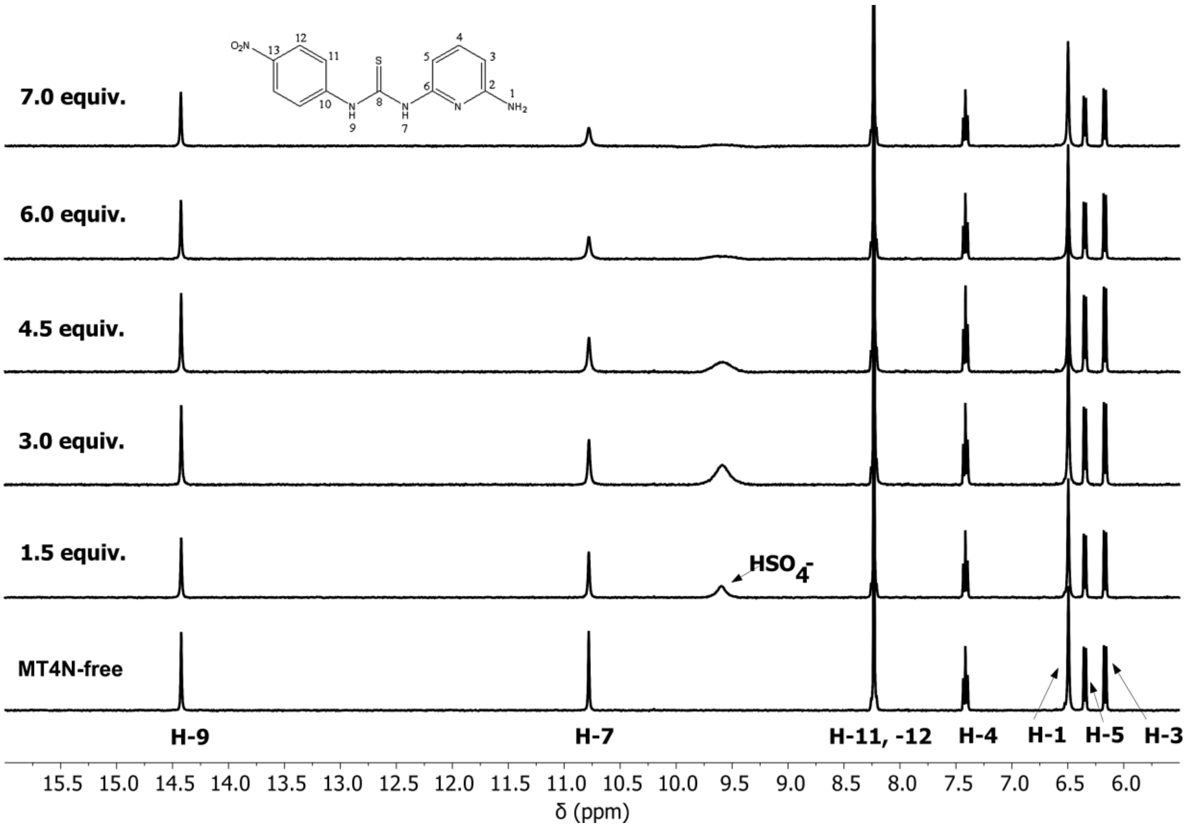

Figure S26. ^{1}H NMR spectra of **MT4N** (3 mM) with increasing concentration of HSO_4^- (0-2.10x10^{-4} M) in DMSO-d_6 at 298 K.

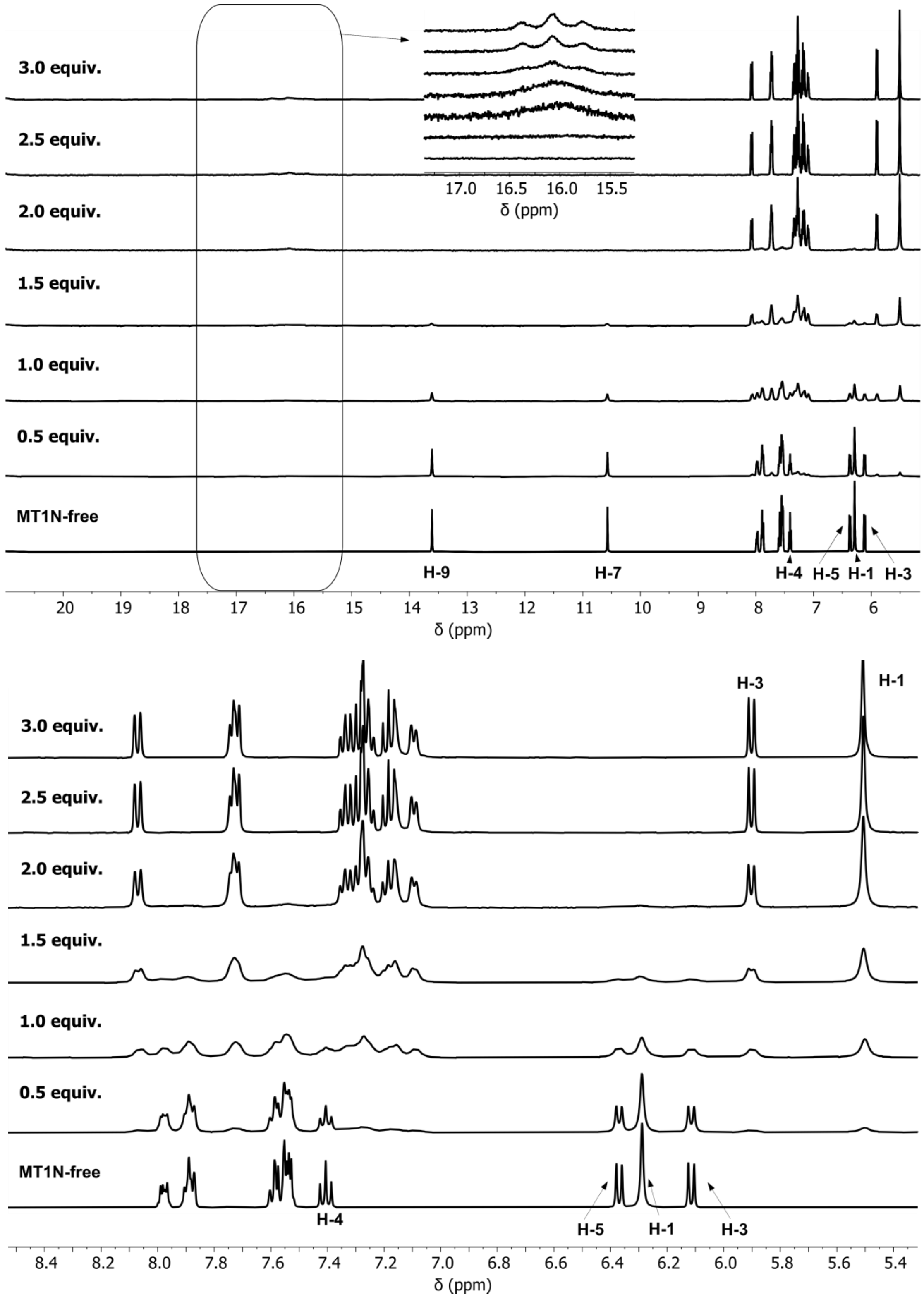

Figure S27. ^{1}H NMR spectra of **MT1N** (3 mM) with increasing concentration of F^- (0-9x10^{-3} M) in DMSO-d_6 at 298 K.

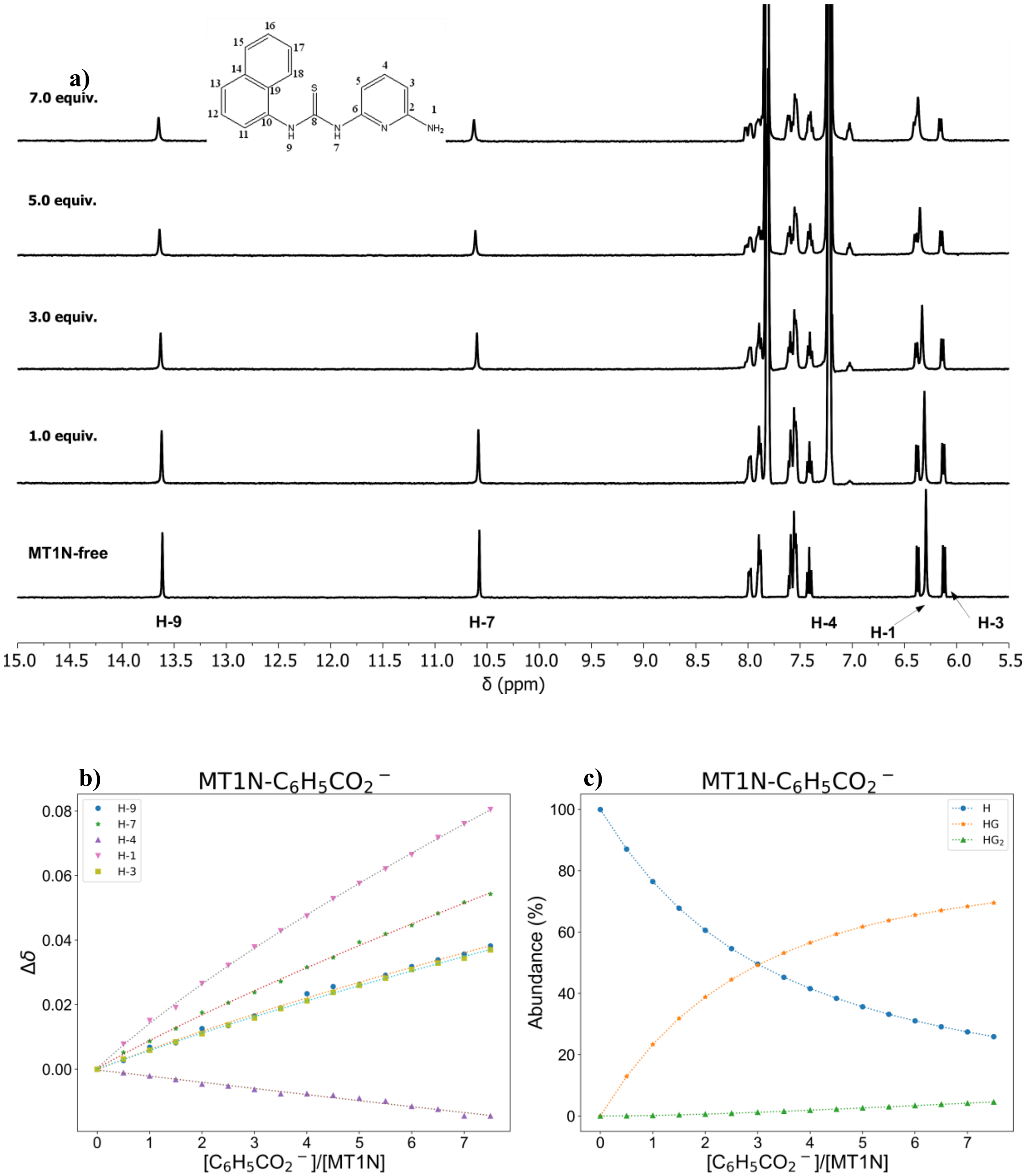

Figure S28. a) Selected spectra from the titration of **MT1N** (3 mM) with $C_6H_5CO_2^-$ (0-$2.25x10^{-4}$ M) in DMSO-d_6 at 298 K. b) Theoretical fit of experimentally measured chemical shift using a 1:2 model by least squares regression. c) Abundance of the different species during the titration, where H = receptor and G = anion guest.

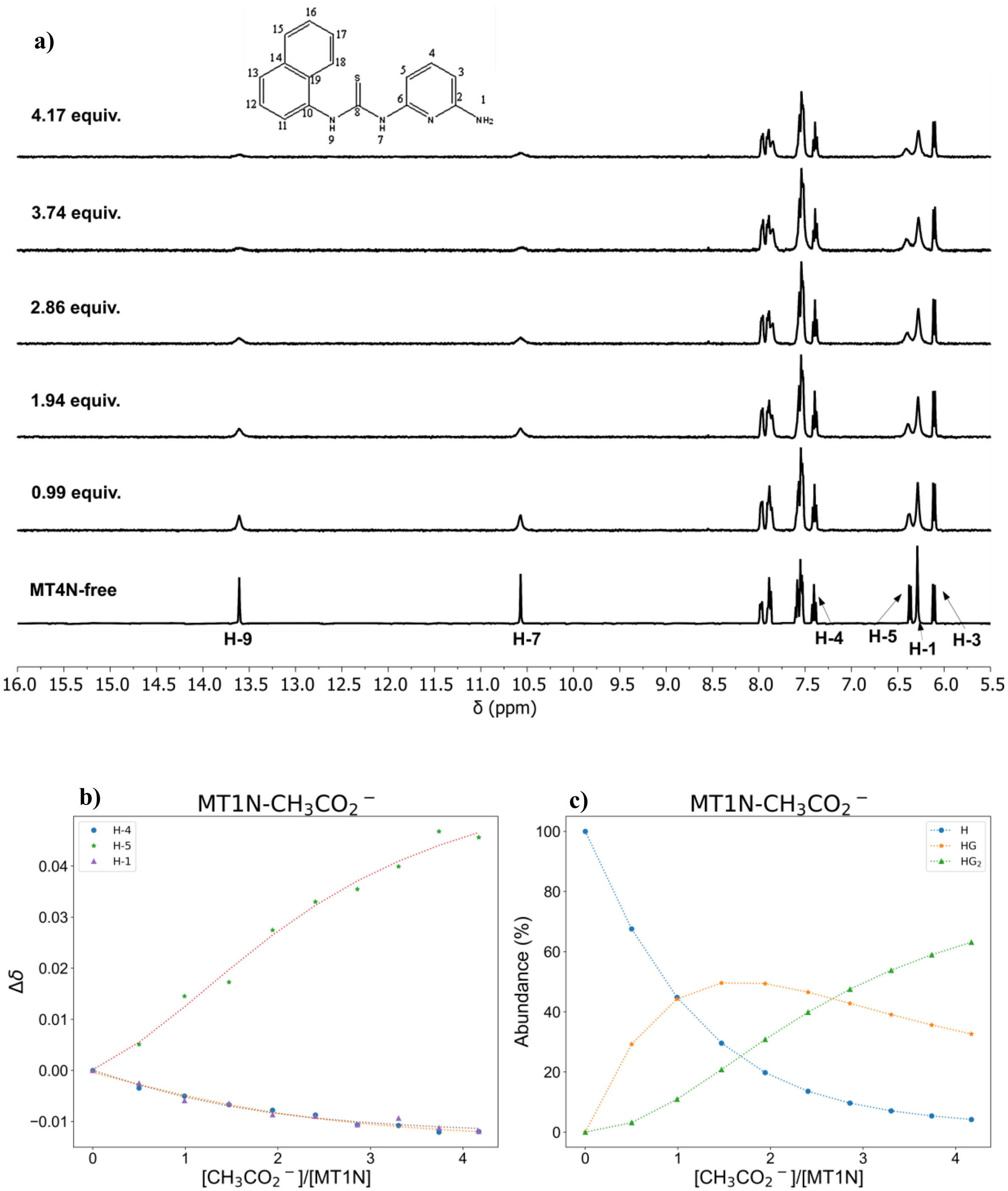

Figure S29. a) Selected spectra from the titration of **MT1N** (3 mM) with $CH_3CO_2^-$ (0-12.51x10^{-3} M) in DMSO-d_6 at 298 K. b) Theoretical fit of experimentally measured chemical shift using a 1:2 model by least squares regression. c) Abundance of the different species during the titration, where H = receptor.

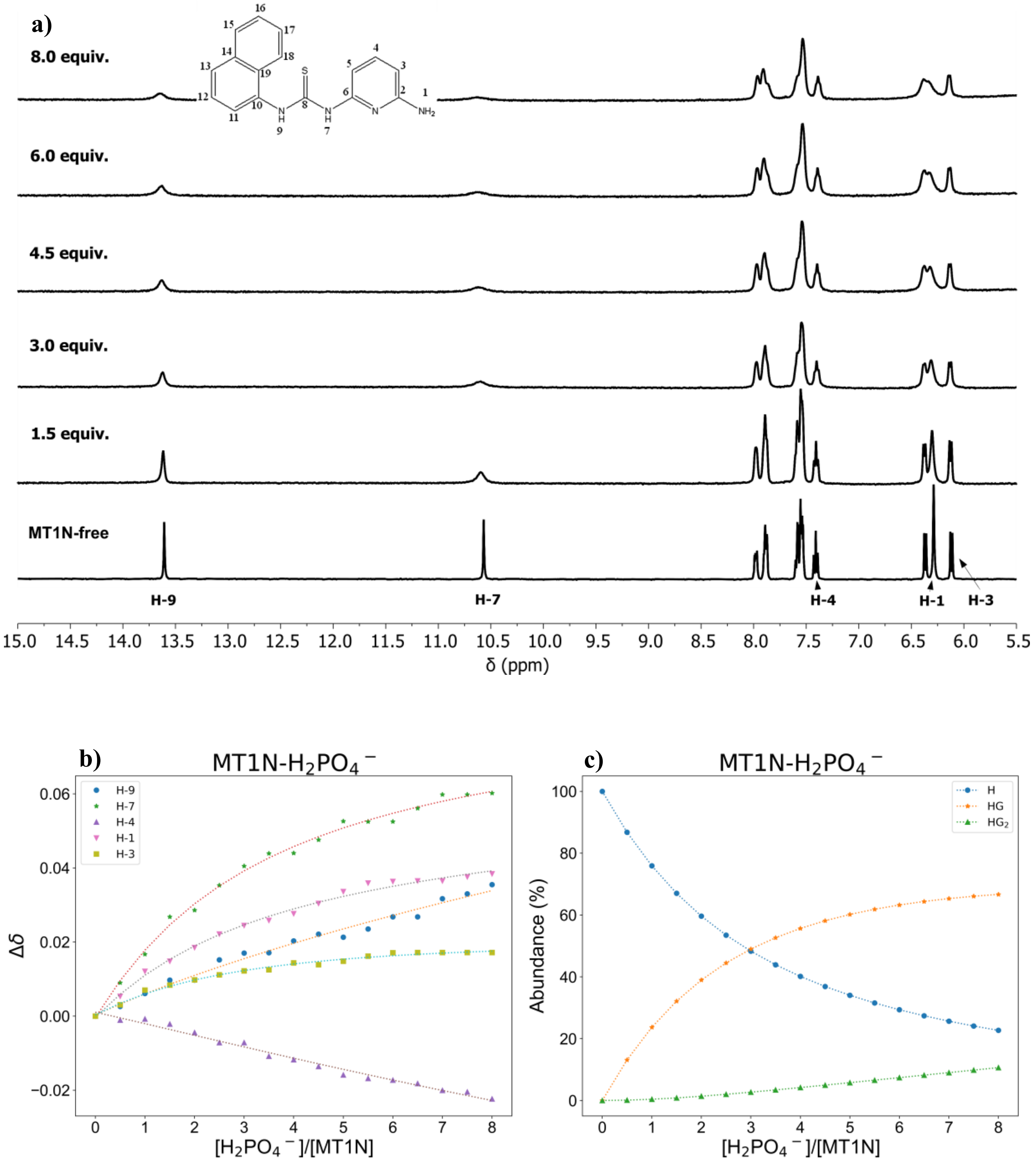

Figure S30. a) Selected spectra from the titration of **MT1N** (3 mM) with $H_2PO_4^-$ (0-2.40x10^{-4} M) in DMSO-d_6 at 298 K. b) Theoretical fit of experimentally measured chemical shift using a 1:2 model by least squares regression. c) Abundance of the different species during the titration, where H = receptor and G = anion guest.

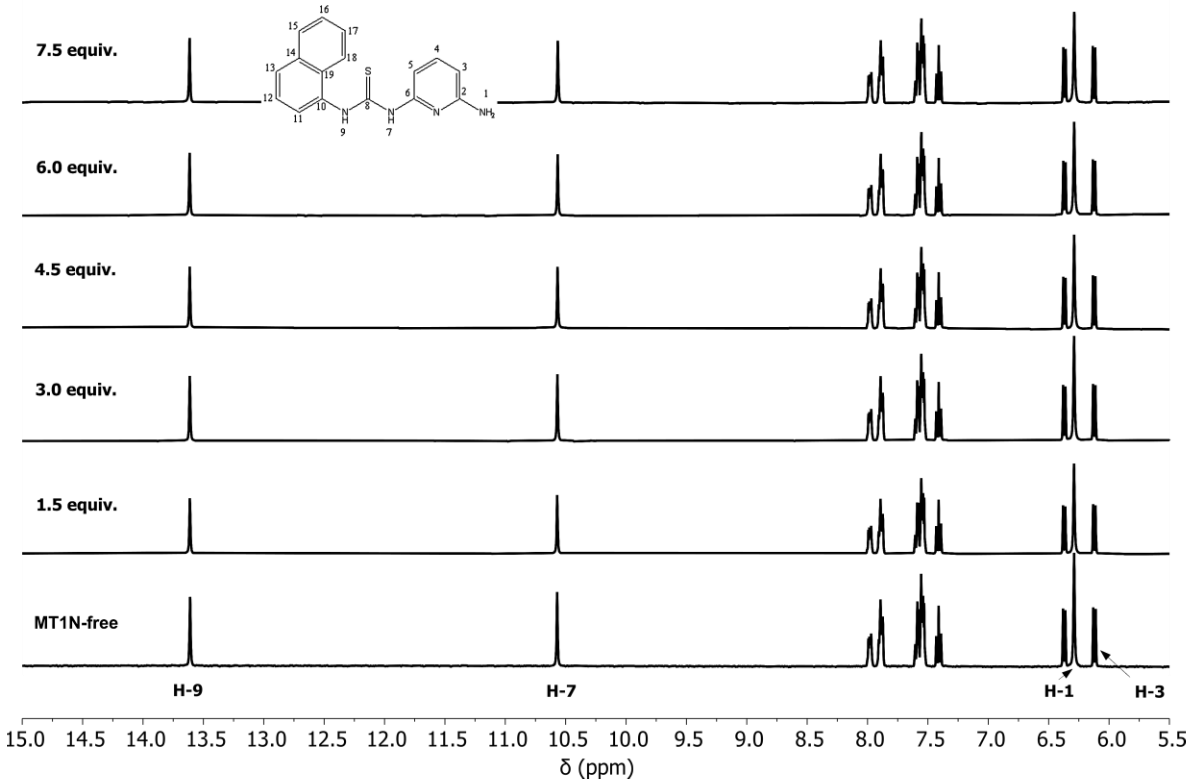

Figure S31. Selected spectra from the titration of **MT1N** (3 mM) with HSO_4^- (0-2.25x10^{-4} M) in DMSO-d_6 at 298 K.

a)

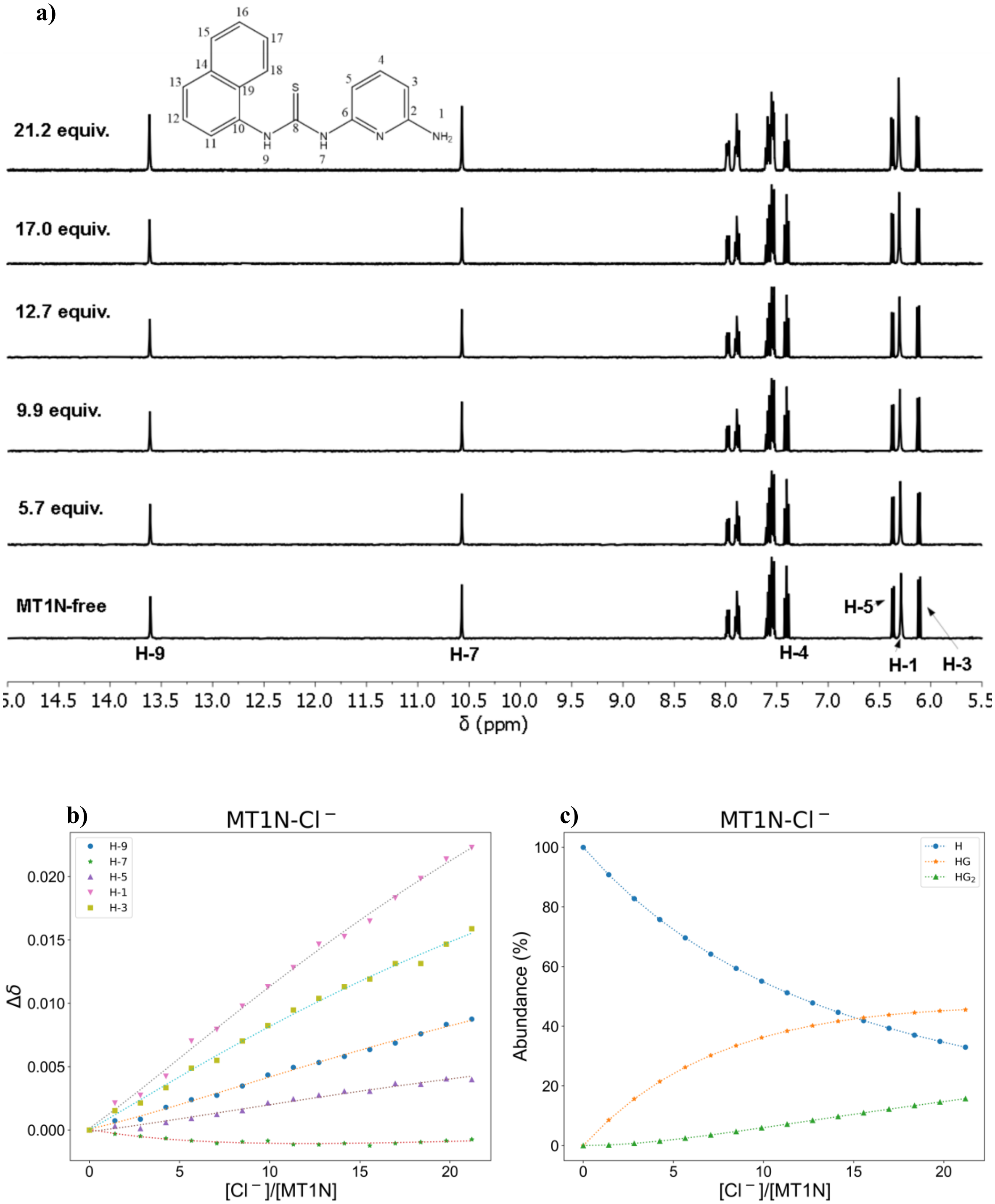

Figure S32. a) Selected spectra from the titration of **MT1N** (3 mM) with Cl^- (0-0.06 M) in DMSO-d_6 at 298 K. b) Theoretical fit of experimentally measured chemical shift using a 1:2 model by least squares regression. c) Abundance of the different species during the titration, where H = receptor and G = anion guest.

Fluorescence

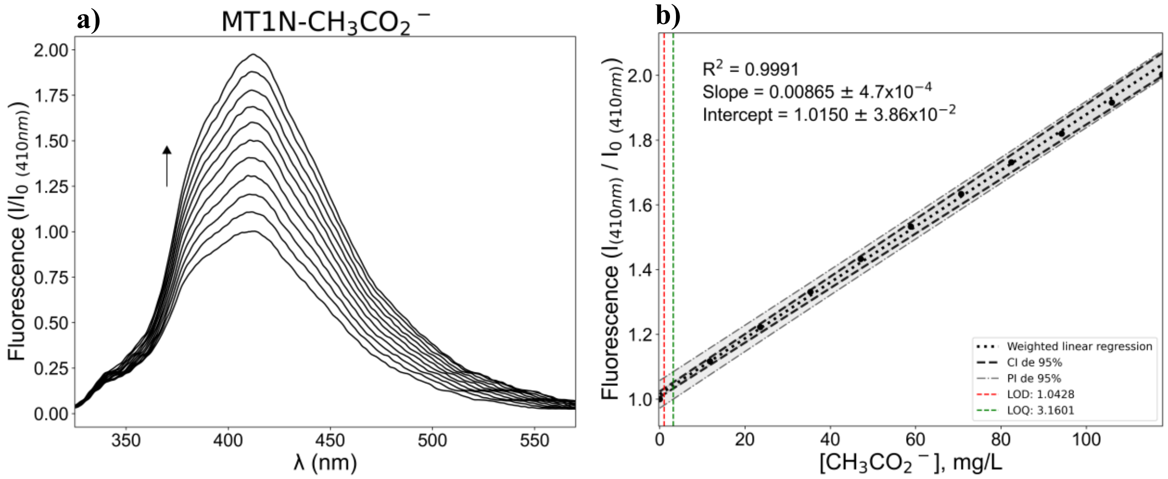

Figure S33. Emission spectra of **MT1N** (1×10^{-5} M) in the presence of increasing concentrations of $CH_3CO_2^-$ ($0\text{-}4\times10^{-4}$ M) in DMSO at 298 K. b) Weighted linear regression of **MT1N**-$CH_3CO_2^-$ λ_{ex} = 342 nm.

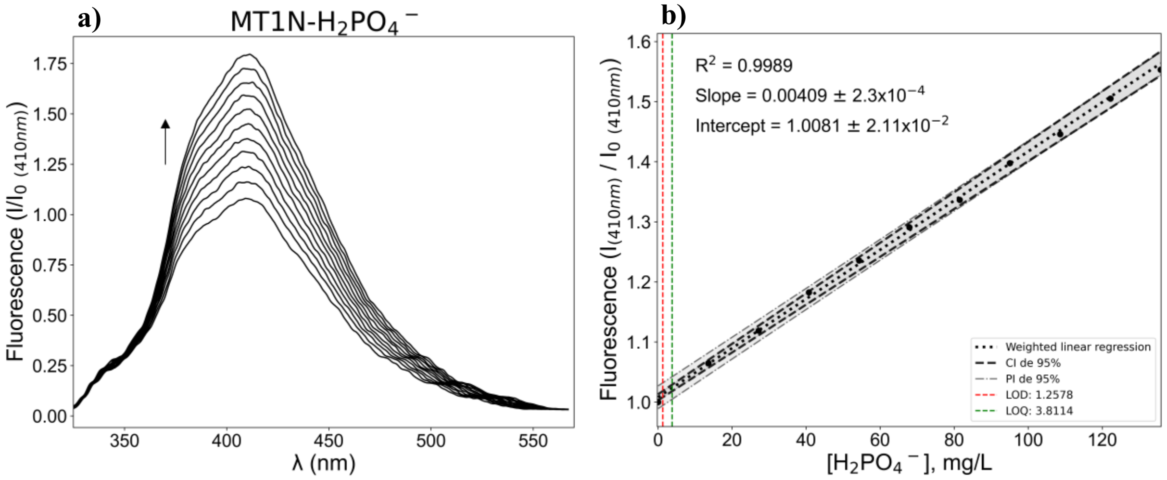

Figure S34. Emission spectra of **MT1N** (1×10^{-5} M) in the presence of increasing concentrations of $H_2PO_4^-$ ($0\text{-}4\times10^{-4}$ M) in DMSO at 298 K. b) Weighted linear regression of **MT1N**-$H_2PO_4^-$. λ_{ex} = 342 nm.

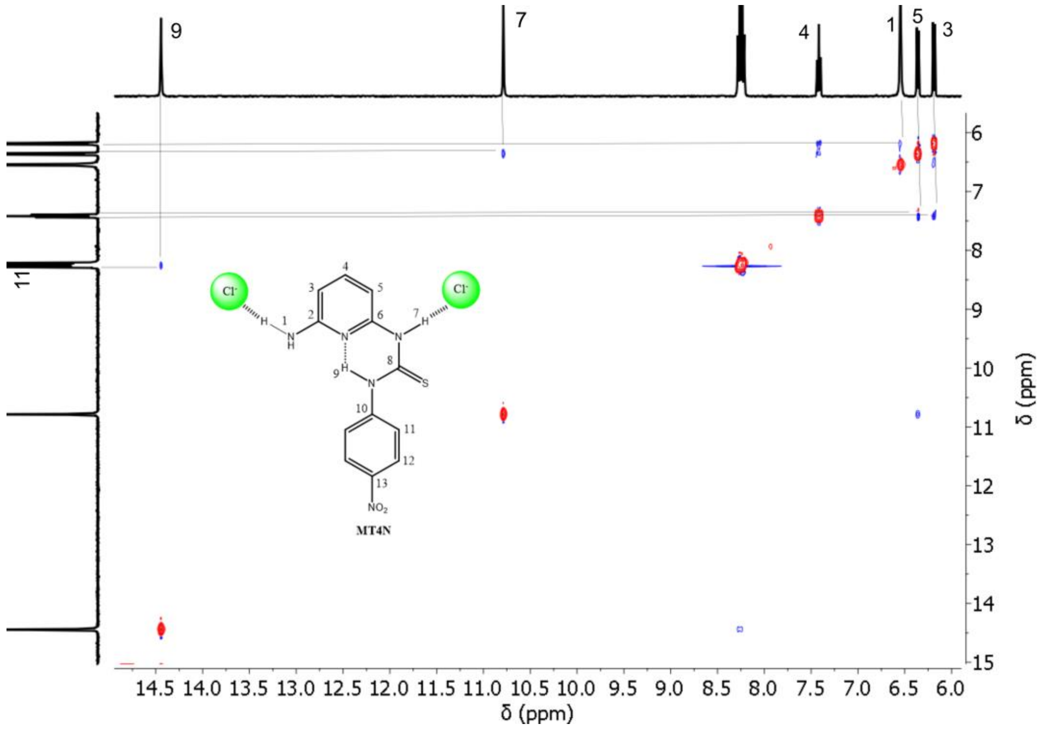

Figure S35. NOESY spectrum of [**MT4N**] = 3mM with 20 equivalents of TBAC in DMSO-d_6 at 289 K.

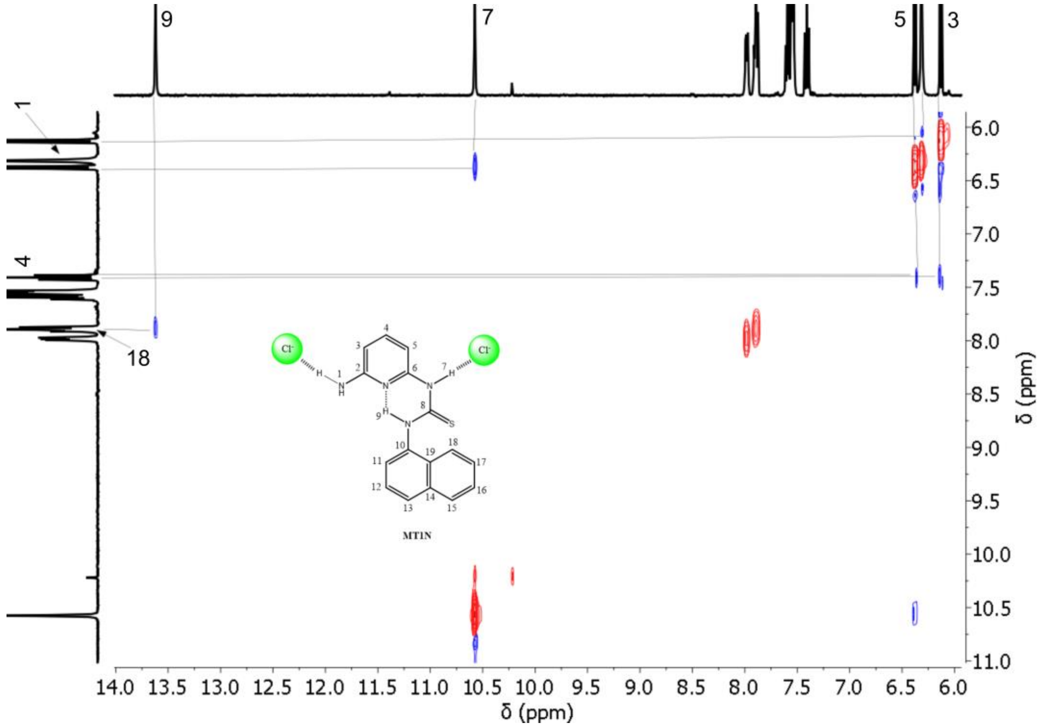

Figure S36. NOESY spectrum of [**MT1N**] = 3mM with 20 equivalents of TBAC in DMSO-d_6 at 289 K.

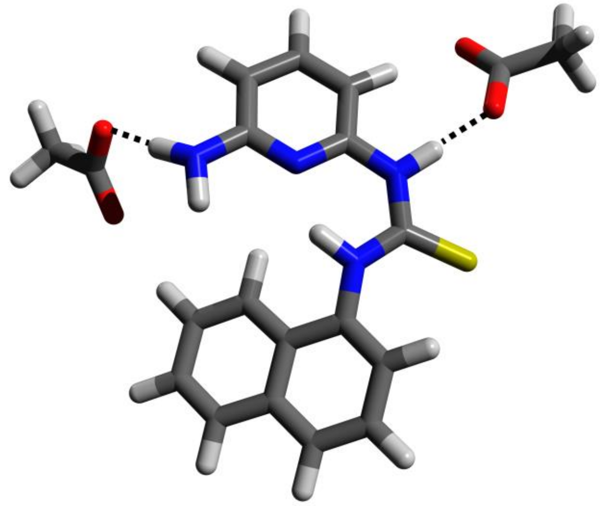

Figure S37. A perspective view of the calculated molecular structure of **MT1N**−$CH_3CO_2^-$ with the B3LYP/6-31G* level of theory, in DMSO.
